# Robust Variable Selection with Exponential Squared Loss for the Spatial Single-Index Varying-Coefficient Model

**DOI:** 10.3390/e25020230

**Published:** 2023-01-26

**Authors:** Yezi Wang, Zhijian Wang, Yunquan Song

**Affiliations:** College of Science, China University of Petroleum, Qingdao 266580, China

**Keywords:** spatial single-index varying-coefficient model, exponential squared loss, variable selection

## Abstract

As spatial correlation and heterogeneity often coincide in the data, we propose a spatial single-index varying-coefficient model. For the model, in this paper, a robust variable selection method based on spline estimation and exponential squared loss is offered to estimate parameters and identify significant variables. We establish the theoretical properties under some regularity conditions. A block coordinate descent (BCD) algorithm with the concave–convex process (CCCP) is composed uniquely for solving algorithms. Simulations show that our methods perform well even though observations are noisy or the estimated spatial mass matrix is inaccurate.

## 1. Introduction

Spatial econometrics is one of the essential branches of econometrics. Its basic content is to consider the spatial effects of variables in regional scientific models. The most widely used spatial econometric model is the spatial autoregressive (SAR) model, first proposed by [1], which has been extensively studied and applied in the fields of economy, finance, and environment.

The SAR model is mainly a parameter model. However, in the practical application, only the parametric model cannot fully explain the complex economic problems and phenomena. Therefore, in order to improve the flexibility and applicability of the spatial econometric model, the non-parametric spatial econometric model has received more attention. Ref. [2] studied the SAR model in the non-parametric frame, obtained the parameter estimators by using the generalized moment estimation, and proved the consistency and asymptotic property of the estimator. The instrumental variable method was used by [3] to study semi-parametric varying-coefficient spatial panel data models with endogenous explanatory variables.

However, for all practical purposes, data may have spatial correlation and spatial heterogeneity simultaneously, which leads to spatial heterogeneity that cannot be fully considered and reflected by the SAR model in the parametric form and the non-parametric SAR model.

The single-index varying-coefficient model is a generalization of the single-index and varying-coefficient models, which can effectively avoid the “curse of dimension” in multidimensional non-parametric regression. Many domestic and foreign researchers have learned this. Refs. [4,5] studied the evaluation of the single-index varying-coefficient model. Ref. [6] constructed the empirical likelihood confidence region of the single-index varying-coefficient model by using the empirical likelihood method; Ref. [7] proposed a new estimated empirical likelihood ratio statistic, obtained maximum likelihood estimators of the model parameters, and proposed a new Profile empirical likelihood ratio, which was shown to be asymptotically close to the standard chi-square distribution.

In addition, selecting significant explanatory variables is one of the most important problems of statistical learning. Some robust regression methods have been proposed, such as quantile regression, composite quantile regression, and modal regression. Ref. [8] presented a new class of robust regression estimators methods for linear models based on exponential square loss. The specific method is as follows: for the linear regression model $y_{i}=X_{i}^{T}\beta +\varepsilon_{i}$, minimize $\sum_{i=1}^{n}\left\{1-\exp\left(-{\left(y_{i}-X_{i}^{T}\beta \right)}^{2}/h\right)\right\}$ this objective function to estimate the regression parameters β, in which h>0 controls the robustness of the estimation. For a large *h*, $1-\exp\left(-r^{2}/h\right)\approx r^{2}/h$. Therefore, the proposed estimation is similar to the least squares estimation in the extreme case. For a small *h*, the value of |r| is large, and the impact on the estimated value is small. Hence, a small value of *h* will limit the influence of outliers on the estimation, thus improving the robustness of estimators. Ref. [8] also pointed out that their method is more robust than the other general robust estimators methods. Ref. [9] made a robust estimation based on exponential square loss for some linear regression models and proposed a data driver to select adjustment parameters. The exponential square loss square is used in data simulation, and positive results were obtained by the method. Ref. [10] suggested a robust variable selection for the high-dimensional single-index varying-coefficient model based on exponential square loss, established and proved the theoretical properties of estimators, and demonstrated the robustness of this method through numerical simulation. Ref. [11] applied exponential square loss to conduct robust structure analysis and variable selection for some linear variable coefficient models and obtained good results.

Inspired by the above article, we introduce the spatial position of the observed objects into a single-index variable coefficient model, and a spatial single-index variable coefficient model is proposed. We also presented a variable selection method for the spatial single-index varying-coefficient model based on spline estimation and the exponential loss function. This method was capable of selecting significant predictors while estimating regression coefficients. The following are the main contributions of this work.

1. We propose a novel model: the spatial single-index varying-coefficient model, which can deal with the spatial correlation and spatial heterogeneity of data at the same time.
2. We construct a robust variable selection method for the spatial single-index varying-coefficient model, which uses exponential square loss function to resist the influence of strong noise and inaccurate spatial weight matrix. Furthermore, we present the BCD (block coordinate descent) algorithm to solve the optimization problem of the objective function.
3. Under reasonable assumptions, we give theoretical properties of this method. In addition, we verify the robustness and effectiveness of the variable selection method through numerical simulation studies. The numerical study shows that the method is more robust than other comparative methods in variable selection and parameter estimation when outliers or noise are presented in the observations.

The rest of this paper is organized as follows. In Section 2, we develop the methodology for variable selection with exponential squared loss and give the theoretical properties of the proposed method in Section 3. In Section 4, we present the related algorithms. The experimental results are carried out in Section 5, and we conclude the paper in Section 6. All of the details of the proofs of the main theorems are collected in the Appendix A.

## 2. Methodology

### 2.1. Model Setup

Consider the following spatial single-index varying-coefficient model:

(1) $$y_{i}=\rho \sum_{j=1}^{n}w_{ij}y_{j}+g_{1}\left(U_{i}^{T}\alpha \right)z_{i1}+g_{2}\left(U_{i}^{T}\alpha \right)z_{i2}+\cdots +g_{q}\left(U_{i}^{T}\alpha \right)z_{iq}+\varepsilon_{i}$$

where $y_{i}$ is the response variable, $z_{i}={\left(z_{i1},z_{i2},\cdots ,z_{iq}\right)}^{T}$ is the *q*-dimensional of the observed variable, and $U_{i}={\left(U_{i1},U_{i2},\cdots ,U_{im}\right)}^{T}$ is the *m*-dimensional spatial location parameter. The n×n matrix of the spatial weights matrix *W* in dimensional space is $w_{ij}$. ρ and $\alpha ={\left(\alpha_{1},\alpha_{2},\cdots ,\alpha_{m}\right)}^{T}$ are the parameters to be estimated. It is natural to suppose that $\varepsilon_{i}$ is independent and subject to a mean value of zero and a variance of $\sigma^{2}$. g(·) is an unknown function. For the identifiability of the model, it is assumed that ∥α∥=1 and the first nonzero element of α is positive.

It can be seen from the model (1) that the spatial single-index varying-coefficient model is a semi-parametric varying-coefficient model, and the unknown function *g* changes with the transformation of geographical location. When $\rho_{}=0$, the model becomes the partial linear single-index varying-coefficient model. When $z_{i1}=1$ and $g_{1}\left(U_{i}^{T}\alpha \right)=U_{i}^{T}\alpha$ while the other g(·)=0, the model becomes the SAR model.

### 2.2. Basis Function Expansion

Since g(·) is unknown, we replace g(·) with its basis function approximations. The specific estimation steps are as follows:

**Step 1.** The initial value $\alpha_{0}$ should be given. This paper uses the method proposed by [12]. We roughly calculate the estimated value of α by the linear regression model:

$$y_{i}=U_{i}^{T}\alpha \;z_{i1}+U_{i}^{T}\alpha \;z_{i2}+\cdots +U_{i}^{T}\alpha z_{iq}+\varepsilon_{i}$$

set the estimated value of α as $\alpha_{0}$, in which $∥\alpha_{0}∥=1$ and the first nonzero element in $\alpha_{0}$ is positive.

**Step 2.** Set $a⩽k_{1}<k_{2}<\cdots <k_{l}⩽b$ as l nodes on the interval [a,b]. By the initial value $\alpha_{0}$, let $t_{i}=U_{i}^{T}\alpha_{0}$, then the radial basis function of degree *p* is

$$\delta \left(t_{i}\right)={\left(1,t_{i},t_{i}^{2},\cdots ,t_{t}^{p-1},{\left|t_{i}-k_{1}\right|}^{2p-1},{\left|t_{i}-k_{2}\right|}^{2p-1},\cdots ,{\left|t_{i}-k_{l}\right|}^{2p-1}\right)}^{T}$$

Suppose that the coefficient of the radial basis function is

$$\gamma_{1-i}={\left(\gamma_{1-i0},\gamma_{1-i1},\cdots ,\gamma_{1-i(p-1)},\gamma_{1-ip},\cdots ,\gamma_{1-i(p+l-1)}\right)}^{T},$$

then, the *s*th unknown function $g_{is}\left(t_{i}\right)\approx \delta {\left(t_{i}\right)}^{T}\gamma_{1-s}$, where i=1,2,⋯,n,s=1,2,⋯,q. Substituting the radial basis function into model (1), we can obtain the following:

(2) $$y_{i}=\rho \sum_{j=1}^{n}w_{ij}y_{j}+z_{i1}\delta {\left(t_{i}\right)}^{T}\gamma_{1-1}+z_{i2}\delta {\left(t_{i}\right)}^{T}\gamma_{1-2}+\cdots +z_{iq}\delta {\left(t_{i}\right)}^{T}\gamma_{1-q}+\varepsilon_{i}$$

Let $Y={\left(y_{1},y_{2},\cdots ,y_{n}\right)}^{T},\gamma_{1}={\left(\gamma_{1-1}^{T},\gamma_{1-2}^{T},\cdots ,\gamma_{1-n}^{T}\right)}^{T},D={\left(D_{1},D_{2},\cdots ,D_{n}\right)}^{T}$, where $D_{i}={\left(z_{i1}\delta {\left(t_{i}\right)}^{T},z_{i2}\delta {\left(t_{i}\right)}^{T},\cdots ,z_{iq}\delta {\left(t_{i}\right)}^{T}\right)}^{T}$, $\varepsilon =\left(\varepsilon_{1},\varepsilon_{2},\cdots \right.,{\left.\varepsilon_{n}\right)}^{T}$, then the matrix form of the model (2) is

(3) $$Y=\rho WY+D\gamma_{1}+\varepsilon$$

As can be seen from model (3), model (1) is transformed from the spatial single-index varying-coefficient model to the classical SAR model under the fitting of the radial basis function. The theory of the SAR model is relatively well-equipped, and the exponential squared loss-based variable selection method for the SAR model is used to estimate the unknown parameters.

### 2.3. The Penalized Robust Regression Estimator

Now, we consider the variable selection for the model (3). To guarantee the model identifiability and to improve the model fitting accuracy and interpretability, we normally assume that the true regression coefficient vector $\alpha^{*}$ is sparse with only a small proportion of nonzeros [13,14]. It is natural to employ the penalized method that simultaneously selects important variables and estimates the values of parameters. The constructed model is recast as follows:  

(4) $$\min_{}L\left(\gamma_{1},\rho \right)=\frac{1}{n}\sum_{i=1}^{n}\phi_{\gamma_{2}}\left(Y_{i}-\rho {\tilde{Y}}_{i}-D_{i}\gamma_{1}\right)+\lambda \sum_{j=1}^{q}P\left(\left|\gamma_{1-j}\right|\right)$$

where λ>0, $\tilde{Y}=WY$, $\sum_{j=1}^{n}P\left(\left|\gamma_{1-j}\right|\right)$ is a penalty term, $\phi_{\gamma_{2}\left(\cdot \right)}$ is the exponential squared loss function: $\phi_{\gamma_{2}\left(t\right)=1-\exp\left(-t^{2}/\gamma_{2}\right)}$, in which $\gamma_{2}$ is the tuning parameter controlling the degree of robustness.

Concerning the choice of the penalty term. The lasso or adaptive lasso penalty could be considered if there is no extra structured information. Assume that $\hat{\gamma_{1}}$ is a root-*n*-consistent estimator for $\gamma_{1}$, for instance, the naive least square estimator ${\hat{\gamma_{1}}}_{\left(\mathrm{ols}\right)}$. Define the weight vector $\eta \in R^{p}$ with $\eta_{j}=1/{\left|{\hat{\gamma }}_{1-j}\right|}^{r}\left(j=1,\ldots ,q\right)$, r>0, and then we set r=1 in this paper as suggested by [15]. An adaptive lasso penalty is described as

(5) $$\sum_{j=1}^{q}P\left(\left|\gamma_{1-j}\right|\right)=\sum_{j=1}^{q}\eta_{j}\left|\gamma_{1-j}\right|.$$

The objective function of penalized robust regression that consists of exponential squared loss and an adaptive lasso penalty is formulated as

(6) $$\min L\left(\gamma_{1},\rho \right)=\frac{1}{n}\sum_{i=1}^{n}\phi_{\gamma_{2}}\left(Y_{i}-\rho {\tilde{Y}}_{i}-D_{i}\gamma_{1}\right)+\lambda \sum_{j=1}^{q}\eta_{j}\left|\gamma_{1-j}\right|$$

The selection of tuning parameter $\gamma_{2}$ and regularization parameter λ is discussed in Section 4.

### 2.4. Estimation of the Variance of the Noise

Set $H={\left(I_{n}-\rho W\right)}^{-1}$, then the variance of the noise is estimated as

(7) $${\hat{\sigma }}^{2}=\frac{1}{n}{\left(Y-HD\gamma_{1}\right)}^{T}{\left(HH^{T}\right)}^{-1}\left(y-HD\gamma_{1}\right),$$

where ρ and $\gamma_{1}$ could be estimated by the solutions of (6). It is pointed out that *H* is a nonsingular matrix, then ${\left(HH^{T}\right)}^{-1}={\left(H^{T}\right)}^{-1}H^{-1}={\left(I_{n}-\rho W\right)}^{T}\left(I_{n}-\rho W\right)$. Let $u=HD\gamma_{1}$, then $u=HD\gamma_{1}={\left(I_{n}-\rho W\right)}^{-1}\left(D\gamma_{1}\right)$ and then ${\hat{\sigma }}^{2}$ defined by (7) can be computed by

(8) $${\hat{\sigma }}^{2}=\frac{1}{n}{∥\left(I_{n}-\rho W\right)\left(Y-u\right)∥}_{2}^{2}$$

## 3. Theoretical Properties

To discuss the theoretical properties, let the parameters $\theta ={\left(\rho ,\gamma_{1}^{T}\right)}^{T}$, with $\theta_{0}$, $\alpha_{0}$ and $g_{0}\left(\cdot \right)$, be the true values of θ, α and g(·). It is generally assumed that $\alpha_{l0}=0$, l=s+1,…,p, and $\alpha_{l0}$, l=1,…,s, are all nonzero parts of $\alpha_{0}$. Moreover, we assume that $g_{j0}=0$, j=d+1,…,q, and $g_{j0}$, j=1,…,d are all nonzero parts of g(·). Set $\phi ={\left(\alpha_{2},\alpha_{3},\cdots ,\alpha_{m}\right)}^{T},\alpha \left(\phi \right)={\left(\sqrt{1-{∥\phi ∥}^{2}},\phi^{T}\right)}^{T}$; the real parameters of $\phi_{0}$ satisfy $∥\phi_{0}∥<1$. Hence, $\alpha_{\varphi }$ is differentiable within the neighborhood of $\phi_{0}$, and the Jacobian matrix is

(9) $$J_{\phi }=\left(\begin{array}{c} -{\left(1-{∥\phi ∥}^{2}\right)}^{-1/2}\phi^{T}\\ I_{m-1} \end{array}\right)$$

Assumption:

(C1) The density function f(t) of Uα is uniformly bounded on T={t=Uα} and far from 0. Furthermore, f(t) is assumed to satisfy the Lipschitz condition of order 1 on T.
(C2) The function $g_{j}\left(t\right),j=1,\ldots ,q$, has bounded and continuous derivatives up to order r(≥2) on *T*, where $g_{j}\left(t\right)$ is the jth components of g(t).
(C3) $E\left(∥U^{6}∥\right)<\infty ,E\left(∥Z^{6}∥\right)<\infty$ and $E\left({\left|\varepsilon \right|}^{6}\right)<\infty$.
(C4) $\left\{\left(y_{i},U_{i},z_{i}\right),1⩽i⩽n\right\}$ is a strictly stationary and strongly mixing sequence with coefficient $\gamma \left(n\right)=O\left(\xi^{n}\right)$, where 0<ξ<1.
(C5) Let $c_{1},\ldots ,c_{K}$ be the interior knots of [a,b], where a=inf{t:t∈T}, b=sup{t:t∈T}. Moreover, we set $c_{0}=a$, $c_{K+1}=b$, $h_{i}=c_{i}-c_{i-1}$, $h=\max\left\{h_{i}\right\}$. Then, a positive constant $C_{0}$ exists such that
  $$\frac{h}{\min\left\{h_{i}\right\}}<C_{0},\;\max\left\{h_{i+1}-h_{i}\right\}=o\left(K^{-1}\right).$$
(C6) Let $b_{n}=\max_{j}\left\{|{\ddot{p}}_{j}\left(\left|\gamma_{1-j0}\right|\right)\right|:\gamma_{1-j0}\neq 0\}$ and then $b_{n}\to 0$ as n→∞. Further, let $\lim_{n\to }\inf_{\infty }$
  $\lim_{\left|\gamma_{1-j}\right|\to }\inf_{0}\lambda_{j}^{-1}\left|{\dot{p}}_{j}\left(\left|\gamma_{1-j}\right|\right)\right|>0$, where j=d+1,…,q.
(C7) $H\left(\rho \right)={\left(I_{n}-\rho W\right)}^{-1}$ is a nonsingular matrix, invertible for any ρ∈Θ, Θ is a compact parameter space, and the absolute row and column sums of H(ρ), $H{\left(\rho \right)}^{-1}$ are uniformly bounded on ρ∈Θ;
(C8) Let
  $$I\left(\phi ,\gamma_{1};\gamma_{2}\right)=\frac{2}{\gamma_{2}}\int G\left(\phi \right)G^{T}\left(\phi \right)e^{-r^{2}/\gamma_{2}}\left(\frac{2r^{2}}{\gamma_{2}}-1\right)dF\left(G,y\right)$$
  where $r=Y-{\left(I_{n}-\rho W\right)}^{-1}D\left(\phi \right)\gamma_{1}=Y-G\left(\phi \right)\gamma_{1},G\left(\phi \right)={\left(I_{n}-\rho W\right)}^{-1}D\left(\phi \right)$. Suppose that $I(\phi ,\gamma_{1};\gamma_{2})$ is negative definite.
(C9) $\Sigma =E\left(GG^{T}\right)$ is positive definite.

Under the above preparations, we give the following sampling properties for our proposed estimators. The following theorem presents the consistency of the penalized exponential squared loss estimators.

**Theorem** **1.** *Assume that conditions C1∼C9 hold and the number of knots $K=O\left(n^{1/(2r+1)}\right)$. Further, we suppose that $\gamma_{2-n}-\gamma_{2-0}=o_{p}\left(1\right)$ for some $\gamma_{2-0}>0$ and $I(\phi_{0},\gamma_{1-0};\gamma_{2-0})$ is negative definite. Then,*

(*i*) *$∥\alpha -\alpha_{0}∥=O_{p}\left(n^{-1/(2r+1)}+a_{n}\right)$;*
(*ii*) *$∥{\hat{g}}_{j}\left(\cdot \right)-g_{j0}\left(\cdot \right)∥=O_{p}\left(n^{-r/(2r+1)}+a_{n}\right)$, for  j=1,…,q,*

*where $a_{n}=\max_{j}\left\{|{\dot{p}}_{j}\left(\left|\gamma_{1-j0}\right|\right)\right|:\gamma_{1-j0}\neq 0\}$, r is defined in condition (C2), and  ${\dot{p}}_{\lambda }\left(\cdot \right)$ represents the first order derivative of $p_{\lambda }\left(\cdot \right)$.*

In addition, we have proved that when some suitable conditions hold, the consistent estimation must be sparse, as described below.

**Theorem** **2.** *Suppose that conditions C1∼C9 hold, and the number of knots $K=O\left(n^{1/(2r+1)}\right)$. We assume that $\sqrt{n}a_{n}=O_{p}\left(1\right)$ and $\sqrt{n}\left(\gamma_{2-n}-\gamma_{2-0}\right)=o_{p}\left(1\right)$. Let*

$$\lambda_{j(\max)}\to 0,\;n^{r/(2r+1)}\lambda_{j(\min)}\to \infty \left(n\to \infty \right).$$

*Then, with probability approaching 1, $\hat{\alpha }$ and $\hat{g}\left(\cdot \right)$ satisfy*

(*i*) *${\hat{\alpha }}_{l}=0$, l=s+1,…,p;*
(*ii*) *${\hat{g}}_{j}\left(\cdot \right)=0$, j=d+1,…,q.*

We then show that the estimators of nonzero coefficients for the parameter components have the same asymptotic distribution as the estimators based on the correct submodel. Set

$$\alpha^{*}={\left(\alpha_{1},\ldots ,\alpha_{s}\right)}^{T},\;g^{*}\left(t\right)={\left(g_{1}^{T}\left(t\right),\ldots ,g_{d}^{T}\left(t\right)\right)}^{T},$$

and let $\alpha_{0}^{*}$ and $g_{0}^{*}\left(t\right)$ be true values of $\alpha^{*}$ and $g^{*}$, respectively. Corresponding covariates are denoted by $U_{i}^{*}$ and $Z_{i}^{*}$, i=1,…,n. Furthermore, let $\Sigma_{2}=\mathrm{cov}\left(\exp\left(-r^{2}/\gamma_{2-0}\right)\frac{2r}{\gamma_{2-0}}G_{i1}\right)$, $\Sigma_{1}=\mathrm{diag}\left\{{\ddot{p}}_{\lambda_{1}}\left(\left|\gamma_{1-01}^{*}\right|\right),\ldots ,{\ddot{p}}_{\lambda_{d}}\left(\left|\gamma_{1-0d}^{*}\right|\right)\right\}$,

$$\begin{array}{c} ∆={\left({\dot{p}}_{\lambda_{1}}\left(\left|\gamma_{1-01}^{*}\right|\right)\mathrm{sign}\left(\gamma_{1-01}^{*}\right),\ldots ,{\dot{p}}_{\lambda_{d}}\left(\left|\gamma_{1-01}^{*}\right|\right)\mathrm{sign}\left(\gamma_{1-01}^{*}\right)\right)}^{T},\\ I_{1}\left(\phi_{01}^{*},\gamma_{1-01}^{*};\gamma_{2-0}\right)=\frac{2}{\gamma_{2-0}}E\left[\exp\left(-r^{2}/\gamma_{2-0}\right)\left(\frac{2r^{2}}{\gamma_{2-0}}-1\right)\right]\left(EG_{i1}G_{i1}^{T}\right). \end{array}$$

The following result presents the asymptotic properties of ${\hat{\alpha }}^{*}$.

**Theorem** **3.** *If the assumptions of Theorem 2 hold, we have*

$$\sqrt{n}\left(I_{1}\left(\phi_{01}^{*},\gamma_{1-01}^{*};\gamma_{2-0}\right)+\Sigma_{1}\right)\left\{{\hat{\alpha }}^{*}-\alpha_{0}^{*}+\left(I_{1}{\left(\phi_{01}^{*},\gamma_{1-01}^{*};\gamma_{2-0}\right)}^{-1}∆\right\}\overset{L}{\to }N\left(0,J_{\phi_{0}^{*}}\Sigma_{2}J_{\phi_{0}^{*}}^{T}\right)\right.$$

*where ‘$\overset{L}{\to }$’ represents the convergence in distribution.*

Theorems 1 and 2 show that the proposed variable selection procedure is consistent, and Theorems 1 and 3 show that the penalized estimators have the oracle property. This demonstrates that if the subset of true zero coefficients are known, the penalty estimators perform well.

## 4. Algorithm

In this section, we talk about a feasible algorithm for the solution of (6). A data-driven procedure for $\gamma_{2}$ and a simple selection method for λ are considered. Moreover, effective optimization algorithms have been composed to solve non-convex and non-differentiable objective functions.

### 4.1. Choice of the Tuning Parameter $\gamma_{2}$

The tuning parameter $\gamma_{2}$ controls the level of robustness and performance of the proposed robust regression estimators. Ref. [16] propose a data-driven procedure to choose $\gamma_{2}$ for ordinary regression. We follow its steps and apply it to the spatial single-index varying-coefficient model. Firstly, a set of tuning parameters is determined to ensure that the proposed penalized robust estimators have an asymptotic breakdown point at 1/2. Then, the tuning parameter is selected with the maximum efficiency.

The whole procedures are presented as follows:

**Step 1.** Initialize $\hat{\rho }=\rho^{\left(0\right)}$ and $\hat{\gamma_{1}}=\gamma_{1}^{\left(0\right)}$. Set $\rho^{\left(0\right)}=\frac{1}{2}$, $\gamma_{1}^{\left(0\right)}$ a robust estimator. The model $Y=\rho WY+D\gamma_{1}+\epsilon$ can be recasted as $Y^{*}=D\gamma_{1}+\epsilon$, where $Y^{*}=Y-\rho WY$.

**Step 2.** Find the pseudo outlier set of the sample:

Let $A_{n}=\left\{\left(D_{1},Y_{1}^{*}\right),\ldots ,\left(D_{n},Y_{n}^{*}\right)\right\}$. Calculate $r_{i}\left(\hat{\gamma_{1}}\right)=Y_{i}^{*}-D_{i}\hat{\gamma_{1}},i=1,\ldots ,n$ and $S_{n}=1.4826\times {\mathrm{median}}_{i}|r_{i}\left(\hat{\gamma_{1}}\right)-{\mathrm{median}}_{j}\left(r_{j}\left(\hat{\gamma_{1}}\right)\right)|$. Then, take the pseudo outlier set $A_{m}=\left\{\left(D_{i},Y_{i}\right):\left|r_{i}\left(\hat{\gamma_{1}}\right)\right|\geq 2.5S_{n}\right\}$, set $m=♯\left\{1\leq i\leq n:\left|r_{i}\left(\hat{\gamma_{1}}\right)\right|\geq \right.\left.2.5S_{n}\right\}$, and $A_{n-m}=A_{n}/A_{m}$.

**Step 3.** Select the tuning parameter $\gamma_{2-n}$: construct $\hat{V}\left(\gamma_{2}\right)={\left\{\hat{I}\left(\hat{\gamma_{1}}\right)\right\}}^{-1}{\tilde{\Sigma }}_{2}{\left\{\hat{I}\left(\hat{\gamma_{1}}\right)\right\}}^{-1}$, in which

$$\hat{I}\left(\hat{\gamma_{1}}\right)=\frac{2}{\gamma_{2}}\left\{\frac{1}{n}\sum_{i=1}^{n}\exp\left(-r_{i}^{2}\left(\hat{\gamma_{1}}\right)/\gamma_{2}\right)\left(\frac{2r_{i}\left(\hat{\gamma_{1}}\right)}{\gamma_{2}}-1\right)\right\}\cdot \left(\frac{1}{n}\sum_{i=1}^{n}D_{i}D_{i}^{T}\right)$$

$${\tilde{\Sigma }}_{2}=\mathrm{Cov}\left\{\exp\left(-r_{1}^{2}\left(\hat{\gamma_{1}}\right)/\gamma_{2}\right)\frac{2r_{1}\left(\hat{\gamma_{1}}\right)}{\gamma_{2}}D_{1},\ldots ,\exp\left(-r_{n}^{2}\left(\hat{\gamma_{1}}\right)/\gamma_{2}\right)\frac{2r_{n}\left(\hat{\gamma_{1}}\right)}{\gamma_{2}}D_{n}\right\}.$$

Let $\gamma_{2-n}$ be the minimizer of $\det(\hat{V}\left(\gamma_{2}\right))$ in the set $G=\{\gamma_{2}:\zeta \left(\gamma_{2}\right)\in \left(0,1\right]\}$, where ζ(·) has the same definition in [8] and det(·) means the determinant operator.

**Step 4.** Update $\hat{\rho }$ and $\hat{\gamma_{1}}$ as the optimal solution of $\min\sum_{i=1}^{n}\phi_{\gamma_{2}}\left(Y_{i}-\rho {\tilde{Y}}_{i}-D_{i}\gamma_{1}\right)$, where $\tilde{Y}=WY$. Repeat step 2 to step 4 until convergence.

It is noted that an initial robust estimator $\gamma_{1}^{\left(0\right)}$ is needed in the initial step above. In practice, we make the estimator of the LAD loss as the initial estimator. In this sense, the selection of $\gamma_{2}$ does not depend on λ basically. Meanwhile, one could also select the two parameters $\gamma_{2}$ and λ jointly by cross-validation as discussed in [8]. Nevertheless, this approach needs huge computation. Moreover, the candidate interval of $\gamma_{2}$ is $\left\{\gamma_{2}:\zeta \left(\gamma_{2}\right)\in \left(0,1\right]\right\}$. Practically, we find the threshold of $\gamma_{2-1}$ subject to $\zeta \left(\gamma_{2-1}\right)=1$. The choice of $\gamma_{2}$ is usually located in the interval of $\left[5\gamma_{2-1},30\gamma_{2-1}\right]$.

### 4.2. Choice of the Regularization Parameter λ and $\eta_{j}$

With regard to the choice of the regularization parameter λ and $\eta_{j}$ in (6), as the parameter λ can be unified with $\eta_{j}$, we set $\lambda_{j}=\lambda \cdot \eta_{j}$. Generally, many methods can be applied to select $\lambda_{j}$, such as AIC, BIC, and cross-validation. To ensure that variable selection is consistent and that the intensive computation can be reduced, we propose the regularization parameter by minimizing a BIC-type objective function as [16]:

(10) $$\sum_{i=1}^{n}\left[1-\exp\left\{-{\left(Y_{i}^{*}-D_{i}\gamma_{1}\right)}^{2}\;/\gamma_{2-n}\right\}\right]+n\sum_{j=1}^{q}\lambda_{j}\left|\gamma_{1-j}\right|-\sum_{j=1}^{q}\log\left(0.5n\lambda_{j}\right)\log\left(n\right)$$

where $Y_{i}^{*}=Y_{i}-\rho W_{n}Y_{i}$. This results in $\lambda_{j}=\frac{\log(n)}{n\left|\gamma_{1-j}\right|}$. $\gamma_{1-j}$ can be easily estimated by the unpenalized exponential squares loss estimator $\tilde{\gamma_{1-j}}$, where the parameter value of $\gamma_{2}$ has been estimated as described in Section 4.1. Note that this simple choice satisfies the conditions $\sqrt{n}{\hat{\lambda }}_{j}\to 0$ for j≤d and $\sqrt{n}{\hat{\lambda }}_{j}\to \infty$ for j>d, with  *d* the number of nonzeros in the true value of $\gamma_{1}$. Thus, the consistent variable selection is ensured by the final estimator.

### 4.3. Block Coordinate Descent (BCD) Algorithm

We seek to compose an effective algorithm to solve the objective function (6). Finding an effective algorithm is difficult because the optimization problem is non-convex and non-differentiable. We embark on using the BCD algorithm proposed by [17] and then overcome the above challenges. The BCD algorithm framework is shown in Algorithm 1 specifically.
**Algorithm 1** The block coordinate descent (BCD) algorithm1.Set initial value for $\gamma_{1}^{0}\in R^{p}$ and $\rho^{0}\in \left(0,1\right)$;2.**repeat** for k=0,1,2,…3.Solve the subproblem about ρ with initial point $\rho^{k}$:
(11)$$\rho^{k+1}\leftarrow \min_{\rho \in [0,1]}L\left(\gamma_{1}^{k},\rho \right)$$4.Solve the subproblem with initial value $\gamma_{1}^{k}$,
(12)$$\min_{\gamma_{1}\in R^{q}}L\left(\gamma_{1},\rho^{k+1}\right)$$
to get a solution $\gamma_{1}^{k+1}$, ensuring that $L\left(\gamma_{1}^{k},\rho^{k+1}\right)-L\left(\gamma_{1}^{k+1},\rho^{k+1}\right)\leq 0$, and  $\gamma_{1}^{k+1}$ is a stationary point of $L\left(\gamma_{1},\rho^{k+1}\right)$.5.**until** convergence.

### 4.4. DC Decomposition and CCCP Algorithm

An elemental observation for problem (12) is that the exponential squared loss function is a DC function, and the lasso or the adaptive lasso penalty function is convex. As a result, problem (12) is a DC programming. It can be solved by the following algorithms.

We first analyze whether the exponential squared loss function $\phi_{\gamma_{2}}\left(t\right)$ can be denoted as the difference of two convex functions:

(13) $$\phi_{\gamma_{2}}\left(t\right):=\left[\phi_{\gamma_{2}}\left(t\right)+v\left(t\right)\right]-v\left(t\right):=u\left(t\right)-v\left(t\right)$$

where $\phi_{\gamma_{2}}\left(t\right)=1-e^{-\frac{t^{2}}{\gamma_{2}}}$, $v\left(t\right)=\frac{1}{3\gamma_{2}^{2}}t^{4}$, $u\left(t\right)=\phi_{\gamma_{2}}\left(t\right)+v\left(t\right)$.

Set

(14) $$\begin{array}{c} J_{\mathrm{vex}}\left(\gamma_{1}\right)=\frac{1}{n}\sum_{i=1}^{n}u\left(Y_{i}-\rho^{k}\langle w_{i},Y\rangle -D_{i}\gamma_{1}\right)+\lambda \sum_{j=1}^{q}P\left(\left|\gamma_{1-j}\right|\right)\\ J_{\mathrm{cav}}\left(\gamma_{1}\right)=\frac{1}{n}\sum_{i=1}^{n}v\left(Y_{i}-\rho^{k}\langle w_{i},Y\rangle -D_{i}\gamma_{1}\right) \end{array}$$

in which u(·), v(·) is defined in (13), $w_{i}$ is in the *i*th row of the weight matrix *W*, and $\sum_{j=1}^{q}P\left(\left|\gamma_{1-j}\right|\right)$ a convex penalty with regard to $\gamma_{1}$. Then, $J_{\mathrm{vex}\;}\left(\cdot \right)$ and $J_{\mathrm{cav}\;}\left(\cdot \right)$ are convex and concave functions, respectively. Subproblem (12) is recast as follows:

(15) $$\min_{\gamma_{1}\in R^{n}}L\left(\gamma_{1},\rho^{k}\right)=J_{\mathrm{vex}}\left(\gamma_{1}\right)+J_{\mathrm{cav}}\left(\gamma_{1}\right),$$

Furthermore, it can be solved by the concave–convex procedure algorithm structure proposed by [18] as shown in Algorithm 2.
**Algorithm 2** The Concave–Convex Procedure1.Initialize $\gamma_{1}^{0}$. Set k=0.2.**repeat** for k=0,1,2,…3.(16)$$\gamma_{1}^{k+1}={\mathrm{argmin}}_{\gamma_{1}}J_{\mathrm{vex}}\left(\gamma_{1}\right)+J_{\mathrm{cav}}^{\prime }\left(\gamma_{1}^{k}\right)\cdot \gamma_{1}$$4.**until** convergence of $\gamma_{1}^{k}$.

We focus on the lasso and the adaptive lasso penalty. Since $J_{\mathrm{cav}\;}^{\prime }\left(\gamma_{1}^{k}\right)\cdot \gamma_{1}$ is linear to $\gamma_{1}$, according to the definition in (15), the objective function of (16) can be expressed as

(17) $$\min_{\gamma_{1}\in R^{q}}\psi \left(\gamma_{1}\right)+\lambda \sum_{i=1}^{q}P\left(\left|\gamma_{1-i}\right|\right),$$

where $\psi (\gamma_{1})$ is a convex and continuously differentiable function, $\sum_{i=1}^{q}P\left(\left|\gamma_{1-i}\right|\right)$ is the lasso penalty, $\sum_{i=1}^{q}\left|\gamma_{1-i}\right|$, or the more general adaptive lasso penalty: $\sum_{i=1}^{q}\eta_{i}\left|\gamma_{1-i}\right|$, $\eta_{i}\geq 0,i=1,\ldots ,q$. Therefore, we can refer to an efficient algorithm ISTA and FISTA proposed by [19] to solve the model with a framework (17) for the lasso penalty. The iterative steps of ISTA is simply $\gamma_{1}^{k}=\Theta_{L}\left(\gamma_{1}^{k-1}\right)$, where *L* is the unknown Lipschitz constant. FISTA is an accelerated version of ISTA that has been shown to have a better convergence rate in theory and practice, proven by [19]. Ref. [17] extended it to solve the model by adaptive lasso penalty and can ensure numerical efficiency.

Now consider solving subproblem (11) to update $\rho_{k}$. Since problem (11) minimizes a function of univariate variable, we employ the classical golden section search algorithm based on parabolic interpolation (see [20] for details).

In accordance with Beck and Teboulle, the value of the iterative function generated by FISTA for solving the subproblem (16) of CCCP converges to the optimal function value at the speed of $O\left(1/k^{2}\right)$, with an iteration step of *k*. The ordinary termination criterion of ISTA and FISTA is $\frac{∥\gamma_{1}^{k}-\gamma_{1}^{k-1}∥}{\max\left\{∥\gamma_{1}^{k}∥,1\right\}}\leq {\mathrm{tol}}_{\gamma_{1}}$, where $tol{}_{\gamma_{1}}$ is a tolerance approaching zero and greater than zero. Under the criterion of either $∥\gamma_{1}^{k}-\gamma_{1}^{k-1}∥\leq \epsilon_{1}$, or $∥L\left(\gamma_{1}^{k}\right)-L\left(\gamma_{1}^{k+1}\right)∥\leq \epsilon_{2}$, Algorithm 1 terminates. Therefore, to obtain an optimal solution of ϵ, the required iterations of the FISTA algorithm are $O(1/\sqrt{\epsilon })$ and the gradient $\nabla \psi (\gamma_{1})$ of (17) is computed for each iteration. Suppose that the BCD algorithm converges with a specified number of iterations and the CCCP algorithm terminates at most *m* times in each iteration. Since O(np) computation is needed to calculate the gradient $\nabla \psi (\gamma_{1})$, the total computational complexity is $O(\mathrm{mnp}/\sqrt{\epsilon })$.

## 5. Simulation Studied

In this section, we conduct numerical studies to illustrate the performance of the proposed method, including the cases of normal data and noisy data.

### 5.1. Simulation Sampling

The data is generated from model (1). We set $\alpha ={\left(\alpha_{1},\alpha_{2},0_{q}\right)}^{T}$, where $\left(\alpha_{1},\alpha_{2}\right)$ generates from a 2-dimensional normal distribution of the mean vector (0.6,0.8) and covariance matrix $0.01\cdot I_{2}$, with $I_{2}$ the unit matrix $\in R^{2\times 2}$, $0_{q}$ is the zero vector of *q* dimension. Set the sample size n∈{25,144,324}, and spatial coefficient ρ is generated by uniform distribution on interval $\left[\rho_{1}-0.1,\rho_{1}+0.1\right]$, where $\rho_{1}\in \left\{0.8,0.5,0.2\right\}$. For comparison’s sake, we also consider ρ=0, which means that there is no spatial dependency, and model (1) changes into the normal single-index varying-coefficient model.

The variable $Y_{n}$ follows $\varepsilon ∼N\left(0,\sigma^{2}I_{n}\right)$, in which $\sigma^{2}$ is generated from a uniform distribution by $\sigma_{1}\in \left\{1,2\right\}$ on interval $\left[\sigma_{1}-0.1,\sigma_{1}+0.1\right]$. We also consider the case when there are outliers in the response. The error term follows a mixed normal distribution $\left(1-\delta_{1}\right)\cdot N\left(0,1\right)+\delta_{1}\cdot N\left(10,6^{2}\right)$, where $\delta_{1}\in \left\{0.01,0.05\right\}$. $z_{ij}$ is independent and randomly taken from the normal distribution N(0,1), and the space weight matrix $W_{n}=I_{R}⊗B_{m}$, where $B_{m}=\left(1/\left(m-1\right)\right)\left(1_{m}\cdot 1_{m}^{T}-I_{m}\right)$, ⊗ is a Kronecker product, and $1_{m}$ is the m-dimensional column vector of ones. We take different values of m=2 and *R*, where *R* = 20,100.

Moreover, we construct the spatial location information, where two-dimensional plane coordinates are used in this paper. Take a square to simulate the geographical area object, set the end point of the lower left corner of the square as the origin, and establish a rectangular coordinate system along the horizontal and vertical directions. Each side is divided into h−1 equal points, and corresponding equal points are connected along the horizontal and vertical axes to form h*h crossing points (including the equal points of each square side). Each crossing point is the geographical location point. Set sampling capacity $n=h^{2}$; then, the geographical location coordinate $U_{i}={\left(u_{i1},u_{i2},\ldots ,u_{iq}\right)}^{T}$ is expressed as:

$$U_{i}={\left(0.5mod\left(i-1,h\right),0.5floor\left(i-1,h\right),0,\ldots ,0\right)}^{T}$$

where *mod* and *floor* are the representations of built-in functions in MATLAB, mod(i−1,h) represents the remainder of i−1 divided by *h*, and floor(i−1,h) represents the integer part of the quotient of i−1 divided by *h*. We set

$$g\left(t\right)={\left(g_{1}\left(t\right),\ldots ,g_{q}\left(t\right)\right)}^{T}={\left(\sin\left(t\right),3t^{2},\frac{1}{6}t,0,\ldots ,0\right)}^{T}$$

**Figure 1:**
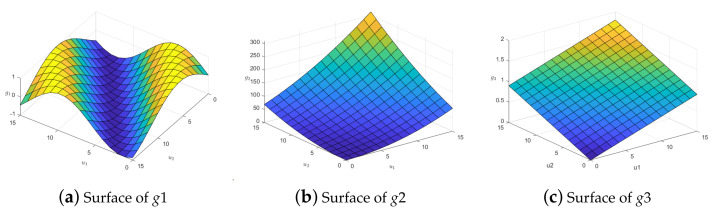
Real surfaces of coefficient functions.

The true surface of the three coefficient functions is shown in Figure 1.

Another important problem of the spatial single-index varying-coefficient model is the estimation of weight matrix *W*. Since $W\in R^{n\times n}$ is composed of the correlation of every two observations, it is usually difficult to obtain an accurate estimation of the weight matrix *W* in practical applications. In order to confirm the effect of inaccurate estimation of the matrix *W*, we randomly remove 30%, 50%, and 80% non-zero weights from each row of the true weight matrix *W*, respectively.

For each case of the simulation experiment, all of the results shown below are averaged over 100 replications to avoid unintended effects. We adopt the node selection method proposed by [12], with step=10 and the number of radial basis functions p=3.

### 5.2. Simulation Results

The evaluation of simulation results is shown as follows. We use the median of squared error (MedSE) proposed by [21]. It is defined as $∥\alpha -\hat{\alpha }{∥}^{2}$ in this paper, where $∥\alpha ∥=\sqrt{\sum_{i=1}^{n}\alpha_{i}^{2}}$, $\alpha =\left(\alpha_{1},\ldots ,\alpha_{n}\right)$, $\hat{\alpha }$ is the estimator of α. The square root of mean deviation (MAISE) is used as the evaluation index for the unknown function. Specifically, $MAISE=mcn^{-1}\sum_{i=1}^{mcn}\sqrt{n^{-1}\sum_{j=1}^{n}{\left(g_{t}\left(U\alpha \right)-\delta {\left(U\hat{\alpha }\right)}^{T}\hat{\gamma_{1-t}}\right)}^{2}},t=1,2,\cdots ,q$, where mcn represents the total simulation times of the model, and *t* represents the *t*th unknown function of the model, $g_{t}\left(\cdot \right)$. The smaller the value of each index, the higher the accuracy of parameter estimation and the better fitting effect of the unknown function.

Table 1 illustrates the results of the estimated coefficient by the spatial single-index varying-coefficient model with q=5, the null penalty term, and Gaussian noise in *y*, where “E”, “S”, and “L” indicate the exponential squared loss, the square loss, and the LAD loss, respectively. It is shown that both of the three loss functions bring nonzero estimates of $\alpha_{1}$ and $\alpha_{2}$, which are close to the true values (the mean of the true values of $\alpha_{1}$ and $\alpha_{2}$ are 0.6, 0.8 resp.). Comparatively, the model with the square loss produces the most accurate estimation. As the sample size increases, all three loss functions bring an accurate estimate of α and $\sigma^{2}$.

Table 2 presents the results of the estimated coefficient by the spatial single-index varying-coefficient model when the dimension is comparatively close to the sample size. Similar results in Table 1 have been observed, except for q=5. As the sample size is not enough compared with the dimension, these results are as expected.

Table 3 illustrates the results of the model when the observations of *y* have outliers. Compared with the square loss model and LAD loss model, the model with exponential square loss shows advantages in parameter estimation in terms of MedSE, especially when the sample size is large.

We list the results of the estimated coefficients with inaccurate weight matrix *W* in Table 4. Compared with the results with normal data (Table 1), the MedSE values increase, and the estimations of $\hat{\rho }$ and ${\hat{\sigma }}^{2}$ become worse for each loss functions in total. Particularly, for removing a certain part (30%, 50%, and 80%) of nonzero weights of the matrix *W*, MedSE increases as the moving nonzeros increase and decreases as the sample size n increases for each of the three loss functions. The exponential squared loss has the lowest MedSE among the three loss functions.

Correspondingly, Table 5, Table 6, Table 7 and Table 8 show the variable selection results compared with other loss functions. The average number of zero coefficients that are correctly chosen is labeled as “Correct”. The label “Incorrect” depicts the average number of nonzero coefficients incorrectly identified as zero. “${\tilde{\ell }}_{1}$”, “$l_{1}$”, and “null” express the adaptive lasso penalty, the lasso penalty, and without penalty term, respectively.

Table 5 shows the variable section results of the lasso and the adaptive lasso regularizer on normal data with q=5. In almost all of the tested cases, the model with the exponential squared loss and the lasso penalty or the adaptive lasso penalty (i.e., $E+l_{1}$, $E+{\tilde{\ell }}_{1}$) identifies more numbers of true zero coefficients (“Correct”) and much lower MedSE.

Similar results have been observed when the sample dimension is close to the sample size, which is presented in Table 6. In the tested cases of n=360,q=200, the model with $\;l_{1}$${\tilde{\ell }}_{1}$ almost correctly identifies all the zero coefficients. The above performance of the proposed exponential squared loss and lasso or adaptive lasso penalty is beyond our expectations.

Table 7 and Table 8 list the variable selections results with noise in the observations and the inaccurate weight matrix. The model with the exponential squared loss and the lasso penalty or the adaptive lasso penalty (i.e., $E+\;l_{1}$, $E+{\tilde{\ell }}_{1}$) identifies many more numbers of true coefficients (“Correct”) and has much lower MedSE. Compared with the results in the normal cases (Table 5), the superiority of $E+\;l_{1}$ and $E+{\tilde{\ell }}_{1}$ is more evident.

**Figure 2:**
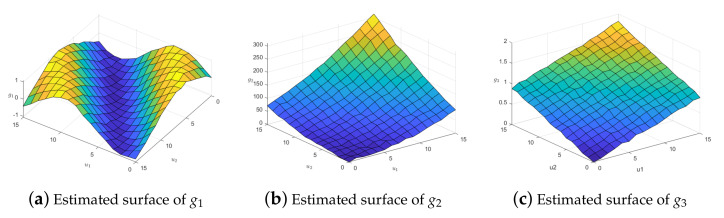
Estimated surfaces of coefficient functions with exponential squared loss.

For the fitting effect diagram of the coefficient function surface, we select the case at the median position in 100 repeated experiments as the standard. Note, we present the situation when h=16 on the normal data. The fitting surfaces of $\hat{g_{1}}$, $\hat{g_{2}}$, and $\hat{g_{3}}$ are shown in Figure 2.

From the fitting effect of each coefficient function, it can be seen that the model has an excellent fitting effect for unknown coefficient functions, which shows that in the case of limited samples, the fitting effect of the spatial single-index varying-coefficient model based on radial basis function and exponential squared loss is excellent. In other cases, the fitting effect of each coefficient function also performs well.

We also present the fitting evaluation index MAISE when h=16,18 on normal data, which is shown in Table 9. It can be seen that with the increase in the total number of spatial objects, the value of the unknown function fitting evaluation index MAISE shows a downward trend. That is, the fitting effect is getting better and better. Similarly, the MAISE value of *y* also shows a downward trend, indicating that for the model as a whole, the relevant data is getting closer to the real data.

**Figure 3:**
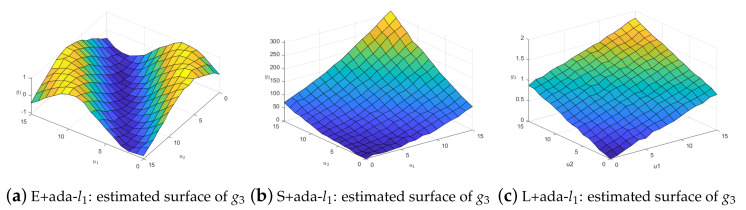
Comparison of $\hat{g_{3}}$ when *y* have outliers.

**Figure 4:**
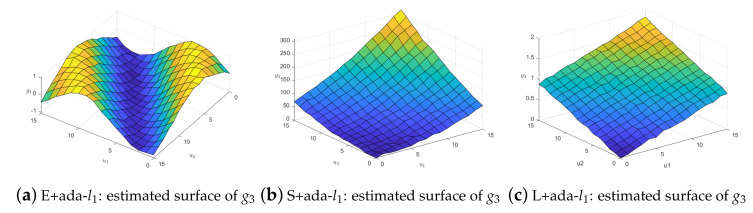
Comparison of $\hat{g_{3}}$ in the case of noisy weighting matrix *w*.

When the observations of *y* have outliers, the coefficient function surface fitting effect is compared. We still select the one in the median of 100 repetitions and take the fitted surface $g_{3}$ as an example. When $\rho_{1}=0.5$, $\sigma_{1}=1$, $\delta_{1}=0.05$, the fitting effect of loss functions with adaptive lasso is shown in Figure 3. This shows that our method performs better. The same conclusion can be conducted in the case of noisy weighting matrix *W*. Figure 4 illustrates the results when we remove 50% nonzero weights.

## 6. Summary

In this paper, we propose a novel model (the spatial single-index varying-coefficient model) and introduce a robust variable selection based on spline estimation and exponential squared loss for the model. The theoretical properties of the proposed estimators are established under reasonable assumptions. We especially design a BCD algorithm equipped with a CCCP procedure for efficiently solving the non-convex and non-differentiable mathematical optimization problem about the variable selection process. Numerical studies show that our proposed method is particularly robust and applicable when observations and the weight matrix are noisy.

## Figures and Tables

**Table 1:** Estimation with no regularizer on normal data (*q* = 5).

	*n* = 25, *q* = 5			*n* = 144, *q* = 5			*n* = 324, *q* = 5		
	E+null	S+null	L+null	E+null	S+null	L+null	E+null	S+null	L+null
$\rho_{1}=0.8$, $\sigma_{1}=1$									
$\alpha_{1}$	0.80	0.61	0.83	0.50	0.66	0.28	0.53	0.62	0.67
$\alpha_{2}$	0.88	0.61	0.75	0.78	0.77	0.66	0.74	0.79	0.96
$\hat{\rho }$	0.80	0.94	0.75	0.90	0.80	0.91	0.88	0.89	0.84
${\hat{\sigma }}^{2}$	0.45	0.78	0.82	0.79	0.72	0.70	0.86	0.71	0.80
MedSE	0.28	0.44	0.70	0.22	0.23	0.42	0.17	0.16	0.19
$\rho_{1}=0.5$, $\sigma_{1}=1$									
$\alpha_{1}$	0.69	0.65	0.64	0.49	0.66	0.28	0.53	0.63	0.67
$\alpha_{2}$	0.82	0.61	0.88	0.78	0.76	0.72	0.74	0.80	0.96
$\hat{\rho }$	0.52	0.61	0.50	0.68	0.40	0.74	0.62	0.65	0.55
${\hat{\sigma }}^{2}$	0.44	0.82	0.71	0.83	0.71	0.74	0.89	0.73	0.82
MedSE	0.22	0.45	0.77	0.22	0.23	0.41	0.17	0.16	0.19
$\rho_{1}=0.2$, $\sigma_{1}=1$									
$\alpha_{1}$	0.67	0.66	0.57	0.51	0.65	0.32	0.53	0.65	0.65
$\alpha_{2}$	0.81	0.59	0.96	0.80	0.75	0.72	0.76	0.81	0.98
$\hat{\rho }$	0.14	0.00	0.27	0.33	0.00	0.50	0.19	0.26	0.16
${\hat{\sigma }}^{2}$	0.44	0.82	0.68	0.87	0.70	0.78	0.91	0.76	0.84
MedSE	0.23	0.49	0.72	0.21	0.23	0.41	0.17	0.16	0.22
$\rho_{1}=0$, $\sigma_{1}=1$									
$\alpha_{1}$	0.68	0.66	0.61	0.51	0.66	0.32	0.53	0.65	0.65
$\alpha_{2}$	0.81	0.60	0.95	0.81	0.73	0.70	0.76	0.81	0.98
$\hat{\rho }$	0.00	0.00	0.22	0.19	0.00	0.36	0.03	0.11	0.04
${\hat{\sigma }}^{2}$	0.44	0.83	0.69	0.88	0.72	0.79	0.91	0.76	0.84
MedSE	0.22	0.47	0.70	0.21	0.25	0.39	0.16	0.16	0.22
$\rho_{1}=0.8$, $\sigma_{1}=2$									
$\alpha_{1}$	0.73	0.77	1.37	0.41	0.79	0.34	0.58	0.63	0.42
$\alpha_{2}$	0.74	0.27	0.64	0.66	0.65	0.55	0.69	0.68	0.96
$\hat{\rho }$	0.86	0.98	0.62	0.95	0.83	0.97	0.93	0.94	0.90
${\hat{\sigma }}^{2}$	1.83	3.26	5.78	3.20	3.08	2.68	3.53	2.89	3.25
MedSE	0.46	1.00	1.95	0.49	0.52	0.90	0.38	0.33	0.48
$\rho_{1}=0.5$, $\sigma_{1}=2$									
$\alpha_{1}$	0.72	0.79	0.66	0.43	0.78	0.23	0.59	0.65	0.42
$\alpha_{2}$	0.74	0.32	0.90	0.68	0.68	0.54	0.72	0.70	1.01
$\hat{\rho }$	0.58	0.68	0.50	0.77	0.29	0.86	0.70	0.74	0.62
${\hat{\sigma }}^{2}$	1.87	3.49	3.05	3.44	3.03	3.00	3.74	3.08	3.45
MedSE	0.46	0.98	1.59	0.48	0.48	0.95	0.38	0.32	0.48
$\rho_{1}=0.2$, $\sigma_{1}=2$									
$\alpha_{1}$	0.76	0.77	0.53	0.46	0.78	0.19	0.60	0.68	0.46
$\alpha_{2}$	0.76	0.35	1.06	0.72	0.65	0.65	0.75	0.72	1.01
$\hat{\rho }$	0.14	0.00	0.39	0.39	0.00	0.61	0.21	0.32	0.23
${\hat{\sigma }}^{2}$	1.88	3.52	3.00	3.68	2.99	3.19	3.88	3.24	3.57
MedSE	0.45	0.97	1.51	0.46	0.50	0.84	0.36	0.32	0.48
$\rho_{1}=0$, $\sigma_{1}=2$									
$\alpha_{1}$	0.77	0.78	0.57	0.47	0.79	0.23	0.59	0.68	0.47
$\alpha_{2}$	0.77	0.34	1.07	0.72	0.64	0.55	0.76	0.72	1.04
$\hat{\rho }$	0.00	0.00	0.31	0.23	0.00	0.52	0.01	0.14	0.07
${\hat{\sigma }}^{2}$	1.88	3.54	3.04	3.74	3.09	3.32	3.90	3.26	3.60
MedSE	0.45	0.97	1.50	0.46	0.52	0.89	0.35	0.33	0.48

**Table 2:** Estimation with no regularizer on normal data when the dimension is close to the sample size.

	n=25, q=20			n=144, q=80			n=324, q=200		
	E+null	S+null	L+null	E+null	S+null	L+null	E+null	S+null	L+null
$\rho_{1}=0.8$, $\sigma_{1}=1$									
$\alpha_{1}$	0.74	0.39	0.24	0.04	0.49	−0.21	0.78	0.72	0.62
$\alpha_{2}$	0.67	0.18	2.81	1.05	0.88	2.15	0.86	0.86	0.71
$\hat{\rho }$	0.84	0.93	0.50	0.54	0.80	0.50	0.80	0.80	0.50
${\hat{\sigma }}^{2}$	0.18	0.53	3.54	0.30	0.26	1.00	0.37	0.41	1.68
MedSE	2.79	2.20	7.92	2.78	1.72	4.56	1.44	1.66	2.21
$\rho_{1}=0.5$, $\sigma_{1}=1$									
$\alpha_{1}$	0.72	0.39	0.10	0.19	0.49	0.06	0.75	0.68	0.56
$\alpha_{2}$	0.65	0.27	1.76	0.94	0.83	1.68	0.84	0.84	0.65
$\hat{\rho }$	0.61	0.66	0.50	0.46	0.54	0.50	0.52	0.53	0.50
${\hat{\sigma }}^{2}$	0.17	0.56	0.44	0.23	0.24	0.47	0.36	0.40	0.72
MedSE	2.87	2.04	3.30	1.57	1.71	2.43	1.40	1.60	1.62
$\rho_{1}=0.2$, $\sigma_{1}=1$									
$\alpha_{1}$	0.70	0.37	0.08	0.17	0.50	0.24	0.75	0.67	0.55
$\alpha_{2}$	0.64	0.40	1.45	0.93	0.79	1.60	0.84	0.85	0.60
$\hat{\rho }$	0.45	0.04	0.50	0.00	0.31	0.50	0.13	0.19	0.50
${\hat{\sigma }}^{2}$	0.18	0.57	0.22	0.22	0.24	0.50	0.36	0.40	0.73
MedSE	3.16	1.71	2.40	1.59	1.82	2.50	1.41	1.59	1.64
$\rho_{1}=0$, $\sigma_{1}=1$									
$\alpha_{1}$	0.71	0.37	0.06	0.20	0.50	0.26	0.75	0.68	0.61
$\alpha_{2}$	0.64	0.38	1.47	0.94	0.80	1.60	0.84	0.85	0.62
$\hat{\rho }$	0.35	0.00	0.50	0.00	0.16	0.50	0.00	0.04	0.50
${\hat{\sigma }}^{2}$	0.18	0.57	0.21	0.23	0.24	0.52	0.36	0.40	0.77
MedSE	3.19	1.76	2.42	1.57	1.81	2.62	1.41	1.60	1.84
$\rho_{1}=0.8$, $\sigma_{1}=2$									
$\alpha_{1}$	0.58	0.08	−0.47	−1.05	0.29	−1.22	0.78	0.68	0.54
$\alpha_{2}$	0.45	−0.57	2.66	1.31	0.87	3.60	0.91	0.71	0.72
$\hat{\rho }$	0.77	0.97	0.50	0.61	0.81	0.50	0.84	0.87	0.50
${\hat{\sigma }}^{2}$	4.18	2.27	12.43	2.20	1.06	4.13	1.63	1.63	6.24
MedSE	8.37	4.41	9.85	5.68	3.59	9.27	2.97	3.24	4.38
$\rho_{1}=0.5$, $\sigma_{1}=2$									
$\alpha_{1}$	0.63	0.11	−0.38	−0.43	0.33	−0.45	0.75	0.68	0.57
$\alpha_{2}$	1.23	−0.23	2.89	1.28	0.78	2.71	0.93	0.71	0.62
$\hat{\rho }$	0.64	0.60	0.50	0.47	0.59	0.50	0.60	0.61	0.50
${\hat{\sigma }}^{2}$	2.33	2.44	1.88	1.76	1.00	2.03	1.73	1.68	3.08
MedSE	5.36	3.80	6.83	4.34	3.55	5.02	2.97	3.26	3.35
$\rho_{1}=0.2$, $\sigma_{1}=2$									
$\alpha_{1}$	0.65	0.13	−0.81	−0.15	0.36	−0.17	0.75	0.69	0.65
$\alpha_{2}$	1.14	−0.04	2.37	0.88	0.74	2.48	0.96	0.72	0.51
$\hat{\rho }$	0.37	0.00	0.50	0.00	0.31	0.50	0.26	0.22	0.50
${\hat{\sigma }}^{2}$	1.89	2.43	0.95	1.95	1.03	2.01	1.91	1.72	3.17
MedSE	5.12	3.43	5.01	3.42	3.66	4.81	3.07	3.30	3.32
$\rho_{1}=0$, $\sigma_{1}=2$									
$\alpha_{1}$	0.64	0.12	−0.80	−0.10	0.35	−0.14	0.76	0.70	0.64
$\alpha_{2}$	0.58	−0.18	2.23	0.88	0.76	2.47	0.93	0.72	0.53
$\hat{\rho }$	0.21	0.00	0.50	0.00	0.15	0.50	0.01	0.01	0.50
${\hat{\sigma }}^{2}$	0.78	2.46	0.97	2.09	1.03	2.07	1.73	1.71	3.24
MedSE	5.91	3.72	4.90	3.42	3.64	4.90	2.98	3.30	3.40

**Table 3:** Estimation with no regularizer when the observations of *y* have outliers.

	n=25, q=5			n=144, q=5			n=324, q=5		
	E+null	S+null	L+null	E+null	S+null	L+null	E+null	S+null	L+null
$\rho_{1}=0.5$, $\sigma_{1}=$1, $\delta_{1}=0.01$									
$\alpha_{1}$	0.71	0.70	0.59	0.52	0.53	0.54	0.52	0.60	0.42
$\alpha_{2}$	0.49	0.76	1.20	0.73	0.94	0.86	0.72	0.66	1.14
$\hat{\rho }$	0.45	0.64	0.50	0.67	0.54	0.51	0.59	0.49	0.55
${\hat{\sigma }}^{2}$	0.80	0.82	0.73	0.87	0.86	0.98	1.02	1.09	0.91
MedSE	0.46	0.34	0.51	0.30	0.25	0.21	0.18	0.22	0.35
$\rho_{1}=0.5$, $\sigma_{1}=2$, $\delta_{1}=0.01$									
$\alpha_{1}$	1.06	0.69	0.64	0.45	0.40	0.45	0.59	0.61	0.35
$\alpha_{2}$	0.14	0.86	1.58	0.62	1.28	0.80	0.71	0.54	1.38
$\hat{\rho }$	0.48	0.69	0.66	0.76	0.59	0.70	0.67	0.52	0.63
${\hat{\sigma }}^{2}$	3.84	3.24	2.82	3.72	3.48	3.98	4.21	4.46	3.79
MedSE	1.52	0.70	1.07	0.55	0.63	0.56	0.39	0.40	0.67
$\rho_{1}=0.5$, $\sigma_{1}=1$, $\delta_{1}=0.05$									
$\alpha_{1}$	0.88	1.01	0.67	0.57	0.74	0.39	0.42	0.58	0.58
$\alpha_{2}$	0.43	0.72	1.01	0.56	0.78	1.00	0.65	0.51	1.05
$\hat{\rho }$	0.60	0.75	0.50	0.75	0.85	0.60	0.67	0.74	0.65
${\hat{\sigma }}^{2}$	2.64	3.72	4.32	3.75	3.02	4.44	4.04	4.64	3.83
MedSE	0.67	0.59	1.07	0.73	0.45	0.47	0.35	0.48	0.28
$\rho_{1}=0.5$, $\sigma_{1}=2$, $\delta_{1}=0.05$									
$\alpha_{1}$	1.09	1.00	0.74	0.56	0.61	0.61	0.48	0.61	0.41
$\alpha_{2}$	0.23	0.83	1.58	0.43	1.13	0.73	0.64	0.40	1.42
$\hat{\rho }$	0.39	0.76	0.50	0.77	0.82	0.50	0.69	0.69	0.64
${\hat{\sigma }}^{2}$	5.02	6.17	6.23	6.13	5.67	7.62	7.08	7.94	6.33
MedSE	0.98	0.83	1.61	0.87	0.59	0.72	0.46	0.64	0.67

**Table 4:** Estimation with no regularizer with noisy weighting matrix *w*.

	n=25, q=5			n=144, q=5			n=324, q=5		
	E+null	S+null	L+null	E+null	S+null	L+null	E+null	S+null	L+null
Remove 30% nonzero weights									
$\alpha_{1}$	0.59	0.58	0.17	0.43	0.32	0.47	0.55	0.65	0.46
$\alpha_{2}$	0.59	0.97	1.63	0.82	0.80	0.97	0.73	0.78	1.12
$\hat{\rho }$	0.61	0.55	0.52	0.70	0.57	0.50	0.65	0.54	0.53
${\hat{\sigma }}^{2}$	1.05	1.13	0.99	1.08	1.08	1.03	1.14	1.09	1.22
MedSE	0.48	0.45	1.10	0.35	0.33	0.49	0.20	0.25	0.25
Remove 50% nonzero weights									
$\alpha_{1}$	0.67	0.57	0.16	0.44	0.30	0.31	0.52	0.66	0.54
$\alpha_{2}$	0.61	0.90	1.48	0.73	0.81	1.15	0.74	0.79	1.10
$\hat{\rho }$	0.54	0.48	0.50	0.64	0.49	0.41	0.57	0.49	0.49
${\hat{\sigma }}^{2}$	1.07	1.17	0.97	1.11	1.11	1.09	1.18	1.12	1.25
MedSE	0.41	0.26	1.12	0.34	0.37	0.64	0.19	0.27	0.31
Remove 80% nonzero weights									
$\alpha_{1}$	0.68	0.63	0.26	0.46	0.33	0.24	0.58	0.70	0.48
$\alpha_{2}$	0.59	0.94	1.29	0.71	0.82	1.31	0.77	0.78	1.19
$\hat{\rho }$	0.40	0.34	0.51	0.52	0.34	0.37	0.42	0.33	0.36
${\hat{\sigma }}^{2}$	1.15	1.30	0.96	1.26	1.24	1.14	1.34	1.26	1.40
MedSE	0.50	0.40	0.89	0.40	0.40	0.78	0.20	0.29	0.33

**Table 5:** Variable section with regularizer on normal data (q=5), E: the exponential loss; S: the square loss; L: the LAD loss; $\;l_{1}$: the lasso penalty; and ${\tilde{\ell }}_{1}$: the adaptive lasso penalty.

	n=25, q=5						n=324, q=5					
	$E+\;l_{1}$	$E+{\tilde{\ell }}_{1}$	$S+\;l_{1}$	$S+{\tilde{\ell }}_{1}$	$L+\;l_{1}$	$L+{\tilde{\ell }}_{1}$	$E+\;l_{1}$	$E+{\tilde{\ell }}_{1}$	$S+\;l_{1}$	$S+{\tilde{\ell }}_{1}$	$L+\;l_{1}$	$L+{\tilde{\ell }}_{1}$
$\rho_{1}=0.8$, $\sigma_{1}=1$												
Correct	4.00	5.00	4.00	5.00	0.00	3.00	5.00	5.00	5.00	5.00	5.00	5.00
Incorrect	0.00	0.00	0.00	0.00	0.00	0.00	0.00	0.00	0.00	0.00	0.00	1.00
$\hat{\rho }$	0.99	0.86	0.86	0.97	0.73	0.82	0.89	0.88	0.88	0.89	0.89	0.92
MedSE	0.42	0.37	0.44	0.36	1.43	0.54	0.14	0.14	0.20	0.16	0.21	0.24
$\rho_{1}=0.5$, $\sigma_{1}=1$												
Correct	4.00	4.00	3.00	5.00	5.00	4.00	5.00	5.00	5.00	5.00	5.00	5.00
Incorrect	0.00	0.00	0.00	0.00	0.00	0.00	0.00	0.00	0.00	0.00	0.00	1.00
$\hat{\rho }$	0.52	0.57	0.58	0.81	0.51	0.46	0.50	0.58	0.58	0.57	0.56	0.68
MedSE	0.24	0.31	0.45	0.40	0.49	0.43	0.17	0.11	0.15	0.16	0.23	0.22
$\rho_{1}=0.2$, $\sigma_{1}=1$												
Correct	4.00	4.00	3.00	5.00	5.00	3.00	5.00	5.00	5.00	5.00	5.00	5.00
Incorrect	0.00	0.00	0.00	1.00	0.00	0.00	0.00	0.00	0.00	0.00	0.00	1.00
$\hat{\rho }$	0.26	0.38	0.40	0.61	0.37	0.20	0.33	0.35	0.35	0.31	0.35	0.49
MedSE	0.24	0.32	0.47	0.42	0.50	0.52	0.14	0.12	0.16	0.16	0.22	0.21
$\rho_{1}=0$, $\sigma_{1}=1$												
Correct	4.00	4.00	3.00	5.00	5.00	3.00	5.00	5.00	5.00	5.00	5.00	5.00
Incorrect	0.00	0.00	0.00	1.00	0.00	0.00	0.00	0.00	0.00	0.00	0.00	1.00
$\hat{\rho }$	0.00	0.06	0.09	0.30	0.01	0.00	0.00	0.00	0.00	0.00	0.00	0.11
MedSE	0.24	0.31	0.46	0.44	0.56	0.55	0.14	0.13	0.17	0.16	0.17	0.21
$\rho_{1}=0.8$, $\sigma_{1}=2$												
Correct	4.00	2.00	1.00	3.00	0.00	1.00	5.00	5.00	5.00	4.00	2.00	4.00
Incorrect	0.00	0.00	0.00	1.00	0.00	0.00	1.00	0.00	0.00	0.00	0.00	1.00
$\hat{\rho }$	0.88	0.90	0.92	0.99	0.86	0.88	0.94	0.92	0.92	0.94	0.92	0.96
MedSE	0.53	0.67	0.94	0.65	2.09	1.05	0.32	0.29	0.32	0.36	0.45	0.44
$\rho_{1}=0.5$, $\sigma_{1}=2$												
Correct	4.00	2.00	1.00	2.00	3.00	3.00	5.00	5.00	5.00	4.00	2.00	4.00
Incorrect	0.00	0.00	0.00	1.00	1.00	0.00	1.00	0.00	0.00	0.00	0.00	1.00
$\hat{\rho }$	0.45	0.64	0.69	0.89	0.51	0.52	0.66	0.63	0.65	0.64	0.62	0.81
MedSE	0.52	0.67	0.96	0.70	0.99	0.95	0.31	0.29	0.31	0.35	0.46	0.48
$\rho_{1}=0.2$, $\sigma_{1}=2$												
Correct	4.00	2.00	1.00	1.00	2.00	3.00	5.00	5.00	5.00	4.00	2.00	3.00
Incorrect	0.00	0.00	0.00	1.00	1.00	0.00	1.00	0.00	0.00	0.00	0.00	1.00
$\hat{\rho }$	0.03	0.45	0.51	0.76	0.50	0.50	0.38	0.37	0.39	0.34	0.40	0.57
MedSE	0.55	0.69	0.97	0.76	1.13	1.02	0.29	0.30	0.33	0.34	0.47	0.48
$\rho_{1}=0$, $\sigma_{1}=2$												
Correct	4.00	2.00	1.00	1.00	2.00	2.00	5.00	5.00	5.00	4.00	3.00	3.00
Incorrect	0.00	0.00	0.00	1.00	1.00	0.00	1.00	0.00	0.00	0.00	0.00	1.00
$\hat{\rho }$	0.00	0.10	0.18	0.49	0.03	0.41	0.00	0.00	0.00	0.00	0.00	0.21
MedSE	0.51	0.68	0.96	0.82	1.09	1.21	0.28	0.31	0.36	0.34	0.38	0.50

**Table 6:** Variable section with regularizer on normal data when the dimension is close to the sample size, E: the exponential loss; S: the square loss; L: the LAD loss; $\;l_{1}$: the lasso penalty; and ${\tilde{\ell }}_{1}$: the adaptive lasso penalty.

	n=25, q=20						n=324, q=200					
	$E+\;l_{1}$	$E+{\tilde{\ell }}_{1}$	$S+\;l_{1}$	$S+{\tilde{\ell }}_{1}$	$L+\;l_{1}$	$L+{\tilde{\ell }}_{1}$	$E+\;l_{1}$	$E+{\tilde{\ell }}_{1}$	$S+\;l_{1}$	$S+{\tilde{\ell }}_{1}$	$L+\;l_{1}$	$L+{\tilde{\ell }}_{1}$
$\rho_{1}=0.8$, $\sigma_{1}=1$												
Correct	7.00	9.00	5.00	6.00	8.00	16.00	195.00	200.00	187.00	195.00	180.00	187.00
Incorrect	1.00	1.00	0.00	0.00	1.00	0.00	1.00	0.00	0.00	0.00	0.00	1.00
$\hat{\rho }$	0.81	0.82	0.89	0.88	0.58	0.69	0.84	0.87	0.85	0.88	0.65	0.73
MedSE	2.89	1.38	3.38	2.06	1.39	0.56	1.23	0.53	1.52	1.36	1.69	1.53
$\rho_{1}=0.5$, $\sigma_{1}=1$												
Correct	6.00	10.00	5.00	4.00	17.00	13.00	197.00	200.00	192.00	196.00	199.00	197.00
Incorrect	0.00	1.00	0.00	0.00	0.00	0.00	0.00	0.00	0.00	0.00	0.00	1.00
$\hat{\rho }$	0.50	0.25	0.55	0.61	0.51	0.42	0.62	0.55	0.54	0.61	0.50	0.50
MedSE	2.02	1.41	3.38	2.20	0.64	0.76	1.05	0.52	1.43	1.33	0.88	1.00
$\rho_{1}=0.2$, $\sigma_{1}=1$												
Correct	5.00	10.00	5.00	5.00	13.00	14.00	197.00	200.00	191.00	193.00	199.00	198.00
Incorrect	1.00	1.00	0.00	0.00	0.00	0.00	0.00	0.00	1.00	0.00	0.00	1.00
$\hat{\rho }$	0.55	0.00	0.40	0.47	0.50	0.23	0.48	0.34	0.40	0.48	0.50	0.50
MedSE	2.98	1.39	3.47	2.45	0.90	0.74	1.11	0.52	1.41	1.36	0.91	1.02
$\rho_{1}=0$, $\sigma_{1}=1$												
Correct	9.00	11.00	5.00	4.00	13.00	13.00	197.00	200.00	192.00	193.00	200.00	196.00
Incorrect	1.00	1.00	0.00	0.00	0.00	0.00	0.00	0.00	1.00	0.00	0.00	1.00
$\hat{\rho }$	0.55	0.00	0.00	0.12	0.38	0.00	0.13	0.00	0.03	0.16	0.50	0.49
MedSE	1.78	1.24	3.41	2.28	1.04	0.82	1.07	0.52	1.42	1.36	0.92	1.13
$\rho_{1}=0.8$, $\sigma_{1}=2$												
Correct	6.00	6.00	5.00	1.00	5.00	13.00	162.00	172.00	133.00	137.00	156.00	138.00
Incorrect	1.00	0.00	0.00	0.00	1.00	0.00	0.00	1.00	1.00	0.00	0.00	1.00
$\hat{\rho }$	0.81	0.92	1.00	0.98	0.76	0.73	0.94	0.89	0.89	0.94	0.73	0.73
MedSE	3.33	3.65	7.30	5.30	2.30	0.92	2.28	1.84	2.99	2.71	2.30	2.78
$\rho_{1}=0.5$, $\sigma_{1}=2$												
Correct	8.00	6.00	4.00	1.00	9.00	9.00	160.00	173.00	134.00	135.00	185.00	173.00
Incorrect	1.00	1.00	0.00	0.00	0.00	0.00	0.00	1.00	1.00	0.00	0.00	1.00
$\hat{\rho }$	0.77	0.53	0.91	0.90	0.60	0.47	0.74	0.63	0.61	0.75	0.50	0.50
MedSE	2.52	3.39	7.63	5.95	1.87	1.44	2.34	1.81	2.96	2.77	1.64	1.94
$\rho_{1}=0.2$, $\sigma_{1}=2$												
Correct	7.00	7.00	4.00	1.00	8.00	9.00	162.00	167.00	129.00	130.00	181.00	173.00
Incorrect	1.00	1.00	0.00	0.00	0.00	0.00	0.00	1.00	1.00	0.00	1.00	1.00
$\hat{\rho }$	0.24	0.10	0.68	0.79	0.63	0.15	0.53	0.46	0.46	0.55	0.50	0.50
MedSE	2.87	3.42	7.62	5.98	1.84	1.47	2.33	1.83	2.97	2.81	1.72	1.99
$\rho_{1}=0$, $\sigma_{1}=2$												
Correct	8.00	6.00	4.00	1.00	9.00	9.00	144.00	172.00	131.00	131.00	178.00	171.00
Incorrect	1.00	0.00	0.00	0.00	0.00	0.00	0.00	1.00	1.00	0.00	1.00	1.00
$\hat{\rho }$	0.00	0.00	0.32	0.54	0.50	0.00	0.35	0.00	0.00	0.26	0.50	0.50
MedSE	2.98	3.60	7.65	5.70	1.95	1.58	2.73	1.83	2.99	2.86	1.87	2.11

**Table 7:** Variable selection with regularizer when the observations *y* have outliers, E: the exponential loss; S: the square loss; L: the LAD loss; $\;l_{1}$: the lasso penalty; and ${\tilde{\ell }}_{1}$: the adaptive lasso penalty.

	n=25, q=5						n=324, q=5					
	$E+\;l_{1}$	$E+{\tilde{\ell }}_{1}$	$S+\;l_{1}$	$S+{\tilde{\ell }}_{1}$	$L+\;l_{1}$	$L+{\tilde{\ell }}_{1}$	$E+\;l_{1}$	$E+{\tilde{\ell }}_{1}$	$S+\;l_{1}$	$S+{\tilde{\ell }}_{1}$	$L+\;l_{1}$	$L+{\tilde{\ell }}_{1}$
$\rho_{1}=0.5$, $\sigma_{1}=1$, $\delta_{1}=0.01$												
Correct	4.00	4.00	4.00	5.00	4.00	3.00	5.00	5.00	5.00	5.00	5.00	5.00
Incorrect	0.00	0.00	0.00	1.00	0.00	0.00	0.00	0.00	0.00	0.00	1.00	0.00
$\hat{\rho }$	0.77	0.64	0.63	0.77	0.70	0.79	0.74	0.66	0.66	0.70	0.64	0.61
MedSE	0.48	0.32	0.48	0.36	0.62	0.53	0.14	0.14	0.17	0.18	0.31	0.30
${\hat{\sigma }}^{2}$												
$\rho_{1}=0.5$, $\sigma_{1}=2$, $\delta_{1}=0.01$												
Correct	3.00	1.00	2.00	3.00	1.00	1.00	5.00	5.00	5.00	5.00	3.00	3.00
Incorrect	0.00	0.00	0.00	0.00	2.00	0.00	0.00	0.00	0.00	0.00	1.00	0.00
$\hat{\rho }$	0.58	0.47	0.52	0.71	0.51	0.76	0.57	0.56	0.58	0.64	0.50	0.50
MedSE	0.61	0.97	0.93	0.79	1.19	1.17	0.18	0.35	0.35	0.34	0.66	0.63
$\rho_{1}=0.5$, $\sigma_{1}=1$, $\delta_{1}=0.05$												
Correct	1.00	4.00	3.00	3.00	0.00	3.00	3.00	4.00	4.00	4.00	4.00	5.00
Incorrect	0.00	1.00	1.00	1.00	1.00	1.00	0.00	1.00	0.00	0.00	0.00	0.00
$\hat{\rho }$	0.78	0.73	0.73	0.90	0.77	0.88	0.83	0.75	0.79	0.79	0.83	0.81
MedSE	0.69	0.87	0.92	0.69	1.75	0.66	0.38	0.30	0.35	0.32	0.52	0.22
$\rho_{1}=0.5$, $\sigma_{1}=2$, $\delta_{1}=0.05$												
Correct	1.00	3.00	1.00	3.00	0.00	3.00	5.00	4.00	4.00	4.00	4.00	3.00
Incorrect	0.00	0.00	1.00	1.00	1.00	0.00	0.00	1.00	0.00	0.00	1.00	0.00
$\hat{\rho }$	0.65	0.42	0.41	0.83	0.51	0.79	0.75	0.59	0.64	0.67	0.63	0.57
MedSE	0.96	1.28	1.26	0.74	1.93	0.54	0.36	0.46	0.46	0.46	0.75	0.54

**Table 8:** Variable selection with regularizer and noisy weighting matrix *w*, E: the exponential loss; S: the square loss; L: the LAD loss; $\;l_{1}$: the lasso penalty; and ${\tilde{\ell }}_{1}$: the adaptive lasso penalty.

	n=25, q=5						n=324, q=5					
	$E+\;l_{1}$	$E+{\tilde{\ell }}_{1}$	$S+\;l_{1}$	$S+{\tilde{\ell }}_{1}$	$L+\;l_{1}$	$L+{\tilde{\ell }}_{1}$	$E+\;l_{1}$	$E+{\tilde{\ell }}_{1}$	$S+\;l_{1}$	$S+{\tilde{\ell }}_{1}$	$L+\;l_{1}$	$L+{\tilde{\ell }}_{1}$
Remove 30% nonzero weights												
Correct	4.00	5.00	5.00	5.00	0.00	5.00	5.00	5.00	5.00	5.00	5.00	5.00
Incorrect	0.00	0.00	0.00	0.00	1.00	0.00	1.00	0.00	0.00	0.00	0.00	0.00
$\hat{\rho }$	0.54	0.53	0.50	0.64	0.28	0.48	0.57	0.55	0.55	0.50	0.55	0.56
MedSE	0.50	0.22	0.41	0.32	0.90	0.28	0.16	0.18	0.26	0.16	0.17	0.28
Remove 50% nonzero weights												
Correct	4.00	5.00	2.00	4.00	2.00	4.00	5.00	5.00	5.00	5.00	5.00	5.00
Incorrect	0.00	0.00	0.00	0.00	1.00	0.00	0.00	0.00	0.00	0.00	0.00	0.00
$\hat{\rho }$	0.55	0.36	0.37	0.54	0.16	0.35	0.38	0.41	0.40	0.35	0.42	0.46
MedSE	0.57	0.43	0.63	0.36	0.86	0.40	0.09	0.22	0.29	0.17	0.12	0.28
Remove 80% nonzero weights												
Correct	4.00	5.00	5.00	3.00	0.00	5.00	5.00	4.00	4.00	5.00	4.00	4.00
Incorrect	0.00	0.00	0.00	0.00	1.00	0.00	0.00	0.00	0.00	0.00	0.00	0.00
$\hat{\rho }$	0.37	0.47	0.43	0.89	0.50	0.72	0.52	0.48	0.46	0.37	0.58	0.49
MedSE	0.63	0.35	0.50	0.77	0.91	0.42	0.20	0.48	0.50	0.29	0.36	0.41

**Table 9:** Results of MAISE for the total number of different spatial objects.

	h=16	h=18
$\hat{g_{1}}$	0.0437	0.0388
$\hat{g_{2}}$	0.0542	0.0539
$\hat{g_{3}}$	0.0515	0.0496
$\hat{y}$	0.0566	0.0548

## Data Availability

Not applicable.

## Abbreviations

The main abbreviations used in this work are as follows:

SAR model:	Spatial autoregressive model;
BCD algorithm:	Block-coordinate descent algorithm;
DC function:	Difference between two convex functions;
CCCP:	concave–convex procedure;
ISTA:	Iterative shrinkage-thresholding algorithm;
FISTA:	Fast iterative shrinkage-thresholding algorithm;
MedSE:	Median of squared error;
MAISE:	Square root of mean deviation.

## Appendix A. Proofs

### Appendix A.1. The Related Lemmas

**Lemma** **A1** (Convexity Lemma). *Let $\left\{h_{n}\left(t\right):t\in T\right\}$ be a sequence of random convex functions defined on a convex, open subset T of $R^{d}$. Assume h(t) is a real-valued function on T for which $h_{n}\left(t\right)\to h\left(t\right)$ in probability, for each t∈T. Then, for each compact subset J of t*

$$\underset{t\in J}{\sup}\left|h_{n}\left(t\right)-h\left(t\right)\right|\to 0\;\mathrm{in}\;\mathrm{probability}.\;$$

*The function h(·) is necessary convex on T.*

**Proof** **of** **Lemma A1.** For this well-known convexity lemma, there are many versions of proof, one of which can be referred to [22]. □

**Lemma** **A2.**
*If $g_{j}\left(t\right),j=1,\ldots ,q$, satisfy condition (C2), then there a constant C>0 exists relying only on M such that*

$$\underset{t\in T}{\sup}\left|g_{j}\left(t\right)-\eta^{T}\left(t\right)\gamma_{1-j}\right|\leq CK^{-r}.$$

**Proof** **of** **Lemma A1.** The proof of Lemma A2 is similar to the proof of inference 6.21 in [23]. □

### Appendix A.2. Poof of Main Theorems

**Proof of Theorem 1.** Let

$$\eta =n^{-r/(2r+1)}+a_{n},\;\phi =\phi_{0}+\eta t_{1},\;\gamma_{1}=\gamma_{1-0}+\eta t_{2},\;t={\left(t_{1}^{T},t_{2}^{T}\right)}^{T}.$$

(i) Let

$$Q\left(\phi ,\gamma_{1}\right)=\sum_{i=1}^{n}\exp\left\{-\left(Y_{i}-G_{i}^{T}{\left(\phi \right)}^{2}/\gamma_{1}\right)\right\}$$

We will present that, for any given ϵ>0, a large constant exists *C* such that

(A1) $$P\left\{\underset{∥t∥=C}{\inf}Q\left(\phi_{0}+\eta t_{1},\gamma_{1-0}+\eta t_{2}\right)>Q\left(\phi_{0},\gamma_{1-0}\right)\right\}\geq 1-\epsilon ,$$

where the true value of ϕ. and $\gamma_{1}$ are $\phi_{0}$ and $\gamma_{1-0}$. Let

$$W_{n}\left(\phi ,\gamma_{1}\right)=\sum_{i=1}^{n}\frac{2}{\gamma_{2}}\exp\left\{-{\left(Y_{i}-G_{i}^{T}\left(\phi \right)\gamma_{1}\right)}^{2}/\gamma_{2}\right\}\left\{Y_{i}-G_{i}{\left(\phi \right)}^{T}\gamma_{1}\right\}{\dot{G}}_{i}{\left(\phi \right)}^{T}\gamma_{1}J_{\phi }^{T}U_{i}$$

and

$$V_{n}\left(\phi ,\gamma_{1}\right)=\sum_{i=1}^{n}\frac{2}{\gamma_{2}}\exp\left\{-{\left(Y_{i}-G_{i}^{T}\left(\phi \right)\gamma_{1}\right)}^{2}/\gamma_{2}\right\}\left\{Y_{i}-G_{i}{\left(\phi \right)}^{T}\gamma_{1}\right\}G_{i}\left(\phi \right),$$

where ${\dot{G}}_{i}^{T}\left(\phi \right)\left(\phi_{0}\right)={\left(I_{n}-\rho W\right)}^{-1}\dot{D}\left(\phi \right)$ Let $L_{n}\left(\tau \right)=K^{-1}\left\{\ell_{n}\left(\phi ,\gamma_{1}\right)-\ell_{n}\left(\phi_{0},\gamma_{1-0}\right)\right\}$. Then, through the Taylor expansion and a simple calculation, we obtain

$$\begin{array}{cc} L_{n}\left(\tau \right)= & \frac{1}{K}\left\{\ell_{n}\left(\phi_{0}+\eta \tau_{1},\gamma_{1-0}+\eta \tau_{2}\right)-\ell_{n}\left(\phi_{0},\gamma_{1-0}\right)\right\}\\ \leq & \frac{1}{K}\eta \left(W_{n}{\left(\phi_{0},\gamma_{0}\right)}^{T},V_{n}{\left(\phi_{0},\gamma_{1-0}\right)}^{T}\right){\left(t_{1},t_{2}\right)}^{T}-\frac{1}{2K}{\left(t_{1},t_{2}\right)}^{T}\left[-I\left(\phi_{0},\gamma_{1-0}\right)\right]\left(t_{1},t_{2}\right)n\eta^{2}\left\{1+o_{p}\left(1\right)\right\}\\ \; & -\frac{n}{K}\sum_{j=1}^{d}\left[]p_{\lambda_{j}}\left(\left|\gamma_{1-j0}\right|\right)-p_{\lambda_{j}}\left(\left|\gamma_{1-j0}\right|\right)\right]\\ = & S_{1}+S_{2}+S_{3}+o_{p}\left(1\right). \end{array}$$

Notice that $S_{1}=O_{p}\left(1+n^{r/\left(2r+1\right)a_{n}}\right)∥t∥$ and $S_{2}=O_{p}\left(\sqrt{n}K^{-1}\eta^{2}\right){∥t∥}^{2}=O_{p}\left(1+2n^{r/(2r+1)}a_{n}\right){∥t∥}^{2}$. Hence, though selecting a sufficiently large *C*, $S_{2}$ dominates $S_{1}$ uniformly in ∥t∥=C. Moreover, invoking $p_{\lambda }\left(0\right)=0$, and by the standard argument of the Taylor expansion, we obtain

$$\begin{array}{cc} S_{3} & \leq n\sum_{j=1}^{d}\left[\eta {\dot{p}}_{\lambda_{j}}\left(\left|\gamma_{1-j0}\right|\right)\mathrm{sgn}\left(\gamma_{1-j0}\right)\left|t_{lj}\right|+\eta^{2}{\ddot{p}}_{\lambda_{j}}\left(\left|\gamma_{1-j0}\right|\right){\left|t_{1j}\right|}^{2}\left\{1+o\left(1\right)\right\}\right]\\ \; & \leq \sqrt{s-1}nK^{-1}\eta a_{n}∥t∥+nK^{-1}\eta^{2}b_{n}{∥t∥}^{2} \end{array}$$

Then, it is clear to present that $S_{3}$ is dominated by $S_{2}$ uniformly in ∥u∥=C. Therefore, selecting a sufficiently large *C*, (A.1) holds. Hence, there exist local minimizers $\hat{\phi }$ and $\hat{\gamma_{1}}$ such that

$$\;∥\hat{\gamma_{1}}-\gamma_{1-0}∥=O_{p}\left(\eta \right),∥\hat{\phi }-\phi_{0}∥=O_{p}\left(\eta \right).$$

By calculating, we obtain $∥\hat{\phi }-\phi ∥=O_{p}\left(\eta \right)$, which finishes the proof of (i). (ii) Note that

$$\begin{array}{cc} {∥{\hat{g}}_{j}\left(t\right)-g_{j0}\left(t\right)∥}^{2} & =\int_{T}{\left\{{\hat{g}}_{j}\left(t\right)-g_{j0}\left(t\right)\right\}}^{2}d\;t\\ \; & =\int_{T}{\left\{\delta^{T}\left(t\right){\hat{\gamma }}_{1-j}-\delta^{T}\left(t\right)\gamma_{1-j0}+R_{j}\left(t\right)\right\}}^{2}d\;t\\ \; & \leq 2\int_{T}{\left\{\delta^{T}\left(t\right){\hat{\gamma }}_{1-j}-\delta^{T}\left(t\right)\gamma_{1-j0}\right\}}^{2}dt+2\int_{T}\left\{R_{j}^{2}\left(u\right)\right\}\\ \; & =2{\left({\hat{\gamma }}_{1-j}-\gamma_{1-j0}\right)}^{T}H\left({\hat{\gamma }}_{1-j}-\gamma_{1-j0}\right)+2\int_{T}\left\{R_{j}^{2}\left(t\right)\right\} \end{array}$$

Then, invoking ∥H∥=O(1), a simple calculation shows

(A2) $${\left({\hat{\gamma }}_{1-k}-\gamma_{1-k0}\right)}^{T}H\left({\hat{\gamma }}_{1-k}-\gamma_{1-k0}\right)=O_{p}\left(n^{-2r/(2r+1)}+a_{n}^{2}\right).$$

In addition, it is easy to show that

(A3) $$\int_{T}R_{j}^{2}\left(t\right)dt=O_{p}\left(n^{-2r/(2r+1)}\right).$$

Invoking (A.2) and (A.3), we finish the proof of (ii). □

**Proof of Theorem 2** (i) From $\lambda_{\max}\to 0$, it is easy to show that $a_{n}=0$ for large *n*. Then, by Theorem 1, it is sufficient to show that, for any $\phi_{j}$ which satisfies

$$∥\phi_{j}-\phi_{j0}∥=O_{p}\left(n^{-r/(2r+1)}\right),\;j=1,\ldots ,s-1,$$

and some given small $\epsilon =Cn^{-r/(2r+1)}$ and j=s,…,p−1, when n→∞, with probability approximating to one, we obtain $\partial Q\left(\phi ,\gamma \right)/\partial \phi_{j}>0$ for $0<\phi_{j}<\epsilon$, and $\partial Q\left(\phi ,\gamma \right)/\partial \phi_{j}<0$ for $-\epsilon <\phi_{j}<0$. Let

(A4) $$Q_{n}\left(\phi ,\gamma_{1}\right)=\sum_{i=1}^{n}\exp\left\{-{\left(Y_{i}-G_{i}^{⊤}\left(\phi \right)\gamma_{1}\right)}^{2}/\gamma_{2}\right\}$$

a simple calculation shows that

$$\begin{array}{cc} \frac{\partial \ell (\phi ,\gamma_{1})}{\partial \phi_{j}}= & \frac{\partial Q_{n}\left(\phi ,\gamma_{1}\right)}{\partial \phi_{j}}\\ = & \sum_{i=1}^{n}\frac{2}{\gamma_{2}}\exp\left\{-{\left(Y_{i}-G_{i}^{T}\left(\phi \right)\gamma_{1}\right)}^{2}/\gamma_{2}\right\}\left\{Y_{i}-G_{i}{\left(\phi \right)}^{T}\gamma_{1}\right\}{\dot{G}}_{i}{\left(\phi \right)}^{T}\gamma_{1}e_{\phi_{j}}^{T}U_{i} \end{array}$$

where $e_{\phi_{j}}={\left(-{\left(1-{∥\phi ∥}^{2}\right)}^{-1/2}\phi_{j},0,\ldots ,0,1,0,\ldots ,0\right)}^{T}$ with (j+1)th component 1. Under conditions (C1), (C2), (C3), and Theorem 1, it is easy to present that

$$\frac{\partial \ell (\phi ,\gamma )}{\partial \phi_{j}}=O_{p}\left(n^{-r/(2r+1)}\right).$$

The sign of the derivative is completely determined by that of $\phi_{j}$. Then, $\partial Q\left(\phi ,\gamma_{1}\right)/\partial \phi_{j}>0$ for $0<\phi_{j}<\epsilon$, and $\partial Q\left(\phi ,\gamma_{1}\right)/\partial \phi_{j}<0$ for $-\epsilon <\phi_{j}<0$ hold. This finishes the proof of (i). (ii) Applying the similar arguments as in the proof of (i), we obtain, with probability approximating to one, ${\hat{\gamma_{1}}}_{j}=0,j=d+1,\ldots ,q$. Then, invoking

$$\underset{t}{\sup}∥\delta \left(t\right)∥=O\left(1\right),$$

the result of this theorem is obtained from ${\hat{g}}_{j}\left(t\right)=\delta^{T}\left(t\right){\hat{\gamma }}_{1-j}$. □

**Proof** **of** **Theorem 3.** Though Theorems 1 and 2, we obtain that, as n→∞, with probability approximating to one, ℓ(ϕ, $\gamma_{1})$ reaches the local maximizer at $\left({\hat{\phi }}^{*T},0^{T}\right)$ and ${\left({\hat{\gamma_{1}}}^{*T},0\right)}^{T}$. Let

$$Q_{1n}\left(\phi ,\gamma_{1}\right)=\frac{\partial \ell (\phi ,\gamma_{1})}{\partial \phi^{*}},\;Q_{2n}\left(\phi ,\gamma_{1}\right)=\frac{\partial \ell (\phi ,\gamma_{1})}{\partial \gamma_{1}^{*}}.$$

Then, ${\left({\hat{\phi }}^{*T},0\right)}^{T}$ and ${\left({\hat{\gamma_{1}}}^{*T},0\right)}^{T}$ satisfy

$$\begin{array}{c} \frac{1}{n}Q_{1n}\left({\left({\hat{\phi }}^{*T},0\right)}^{T},{\left({\hat{\gamma_{1}}}^{*T},0\right)}^{T}\right)\\ \;=\frac{-2}{n}\sum_{i=1}^{n}\frac{2}{\gamma_{2}}\exp\left\{-{\left(Y_{i}-G_{i}^{T}\left(\phi \right)\gamma_{1}\right)}^{2}/\gamma_{2}\right\}\left(Y_{i}-G_{i}^{*T}\left({\hat{\phi }}_{0}^{*}\right){\hat{\gamma_{1}}}^{*}\right){\dot{G}}_{i}^{*T}\left({\hat{\phi }}^{*}\right){\hat{\gamma_{1}}}^{*}J_{{\hat{\phi }}^{*}}^{T}U_{i}^{*T}-V_{1}=0 \end{array}$$

where

$$V_{1}={\left({\dot{p}}_{\lambda_{1}}\left(\left|{\hat{\gamma }}_{1-1}\right|\right)\frac{{\hat{\gamma }}_{1-1}^{T}H}{\left|{\hat{\gamma }}_{1-1}\right|},\ldots ,{\dot{p}}_{\lambda_{d}}\left(\left|{\hat{\gamma }}_{1-d}\right|\right)\frac{{\hat{\gamma }}_{1-d}^{T}H}{{\left|{\hat{\gamma }}_{1-d}\right|}_{H}}\right)}^{T}.$$

Applying the Taylor expansion to ${\dot{p}}_{\lambda_{j}}\left(\left|{\hat{\gamma }}_{1-j}\right|\right)$, we have

$${\dot{p}}_{\lambda_{j}}\left(\left|{\hat{\gamma }}_{1-j}\right|\right)={\dot{p}}_{\lambda_{j}}\left(\left|{\hat{\gamma }}_{1-j0}\right|\right)+\left\{{\ddot{p}}_{\lambda_{j}}\left(\left|{\hat{\gamma }}_{1-j0}\right|\right)+o_{p}\left(1\right)\right\}\left({\hat{\gamma }}_{1-j}-\gamma_{1-j0}\right).$$

Furthermore, condition (C6) implies that ${\ddot{p}}_{\lambda_{j}}\left(\left|{\hat{\gamma }}_{1-10}\right|\right)=o_{p}\left(1\right)$, and note that ${\dot{p}}_{\lambda_{j}}\left(\left|{\hat{\gamma }}_{1-10}\right|\right)=0$ as $\lambda_{\max}\to 0$. From Theorems 1 and 2, we have

$${\dot{p}}_{\lambda_{j}}\left(\left|{\hat{\gamma }}_{1-j}\right|\right)\frac{{\hat{\gamma }}_{1-j}^{T}H}{\left|{\hat{\gamma }}_{1-j}\right|}=o_{p}\left({\hat{\gamma_{1}}}^{*}-\gamma_{1-0}^{*}\right)..$$

Therefore, a simple calculation shows that

$$\begin{array}{cc} \frac{1}{n}\sum_{i=1}^{n}\left(Y_{i}-G_{i}^{*T}\left({\hat{\phi }}_{0}^{*}\right){\hat{\gamma_{1}}}^{*}\right)G_{i}^{*}\left({\hat{\phi }}^{*}\right)= & \frac{1}{n}\sum_{i=1}^{n}\left\{\varepsilon_{i}+R^{T}\left(\alpha_{0}^{*T}U_{i}^{*}\right)Z_{i}^{*}-G_{i}^{*T}\left(\phi_{0}^{*}\right)\left({\hat{\gamma_{1}}}^{*}-\gamma_{1-0}^{*}\right)\right.\\ \; & \left.-{\left[G_{i}^{*}\left({\hat{\phi }}_{0}^{*}\right)-G_{i}^{*}\left(\phi_{0}^{*}\right)\right]}^{T}{\hat{\gamma_{1}}}^{*}\right\}\left\{G_{i}^{*}\left(\phi_{0}^{*}\right)+\left[G_{i}^{*}\left({\hat{\phi }}^{*}\right)-G_{i}^{*}\left(\phi_{0}^{*}\right)\right]\right\} \end{array}$$

Let

$$\Lambda_{n}=\frac{1}{n}\sum_{i=1}^{n}G_{i}^{*}\left(\phi_{0}^{*}\right)\left(\varepsilon_{i}+R^{T}\left(U_{i}^{*T}\alpha_{0}^{*}\right)Z_{i}^{*}\right).$$

Then, from conditions (C8) and (C9), Theorem 1, and $\sup_{t}∥\delta \left(t\right)∥=O\left(1\right)$, we obtain

$${\hat{\gamma_{1}}}^{*}-\gamma_{1-0}^{*}={\left[\Phi_{n}+o_{p}\left(1\right)\right]}^{-1}\left\{\Lambda_{n}-\Psi_{n}\left({\hat{\phi }}^{*}-\phi_{0}^{*}\right)\right\}.$$

Thus, we can have

$$\begin{array}{cc} \frac{\ell_{n}\left({\left({\hat{\phi }}^{*T},0\right)}^{T},{\left({\hat{\gamma_{1}}}^{*T},0\right)}^{T}\right)}{\partial \phi_{j}^{*}}= & \frac{\partial Q_{n}\left\{{\left(\phi_{0}^{*T},0\right)}^{T},{\left(\gamma_{1}^{*T},0\right)}^{T}\right\}}{\partial \phi_{j}^{*}}\\ = & \frac{\partial Q_{n}\left\{{\left(\phi_{0}^{*T},0\right)}^{T},{\left(\gamma_{1-0}^{*T},0\right)}^{T}\right\}}{\partial \phi_{j}^{*}}\\ \; & +\sum_{l=1}^{s-1}\left\{\frac{\partial^{2}Q_{n}\left\{{\left(\phi_{0}^{*T},0\right)}^{T},{\left(\gamma_{1-0}^{*T},0\right)}^{T}\right\}}{\partial \phi_{j}^{*}\phi_{l}^{*}}+o_{p}\left(1\right)\right\}\left({\hat{\phi }}_{l}^{*}-\phi_{01}^{*}\right) \end{array}$$

where $Q_{n}\left(\phi ,\gamma_{1}\right)$ is defined in (A.4). As the definition of $J_{\phi_{0}^{*}}$ that $\alpha -\alpha_{0}=J_{\phi_{0}^{*}}\left(\phi -\phi_{0}^{*}\right)+O_{p}\left(n^{-1}\right)$. Since $\sqrt{n}\left(\gamma_{2-n}-\gamma_{2-0}\right)=o_{p}\left(1\right)$, the proof is proved by Slutsky’s lemma and the central limit theorem. This ends with proof of Theorem 3. □

Thus, the proof is completed.
